# Acknowledgement to Reviewers of *Molecules* in 2015

**DOI:** 10.3390/molecules21010131

**Published:** 2016-01-21

**Authors:** Molecules Editorial Office

**Affiliations:** MDPI AG, Klybeckstrasse 64, CH-4057 Basel, Switzerland; molecules@mdpi.com

The editors of *Molecules* would like to express their sincere gratitude to the following reviewers for assessing manuscripts in 2015.

We greatly appreciate the contribution of expert reviewers, which is crucial to the journal’s editorial decision-making process. Several steps have been taken in 2015 to thank and acknowledge reviewers. Good, timely reviews are rewarded with a discount off their next MDPI publication. By creating an account on the submission system, reviewers can access details of their past reviews, see the comments of other reviewers, and download a letter of acknowledgement for their records. In addition, MDPI has launched a collaboration with Publons, a website that seeks to publicly acknowledge reviewers on a per journal basis. This is all done, of course, within the constraints of reviewer confidentiality. Feedback from reviewers shows that most see their task as a voluntary and mostly unseen work in service to the scientific community. We are grateful to our reviewers for the contribution they make.

Aaron, Jean E.
Abagyan, Ruben
Abdulhameed, Mohamed Diwan
Abe, Sumiko
Abenavoli, Ludovico
Abioye, Amos Olusegun
Abou-Hassan, Ali
Abram, Veronika
Abrouk, Michael
Abu-Reidah, Ibrahim M.
Achelle, Sylvain
Acuña, Luis
Adam, Rosa
Adams, Michael
Adhami, Hamid-Reza
Adhikary, Amitava
Adriano, Marocco
Afarinkia, Kamyar
Afonso, Julio Carlos
Agostino, Mark
Agranat, Israel
Aguedo, Mario
Agut, Montserrat
Ahmed, Nisar
Aires, Alfredo
Aisa, Haji Akber
Aizawa, Mineaki
Akagawa, Mitsugu
Akashi, Ryo
Åkermark, Björn
Akine, Shigehisa
Aktories, Klaus
Alarcón, Julio
Alashi, Adeola M.
Albacete, Alfonso
Albini, Angelo
Aldabbagh, Fawaz
Aldrich-Wright, Janice R.
Al-Dujaili, Emad
Aléman, José
Al-Horani, Rami A.
Ali, Mubarak
Alitalo, Anni
Allais, Florent
Allegaert, Karel
Allen, Randy
Allen, William J.
Allenmark, Stig
Almagro, Lorena
Almajano, Mariapilar
Alonsoa, Francisco
Alonso-Monge, Rebeca
Al-Rawi, Jasim
Altemimi, Ammar
Altomare, Cosimo Damiano
Aluko, Rotimi
Alvarez-Builla, Julio
Alvarez-Lorenzo, Carmen
Alvarez-Manzaneda, E.J.
Alvarez-Suarez, José M.
Alvarez-Suarez, José Miguel
Alves, Diego
Alves, Gilberto
Alves, Isabel D.
Aly, Hamed
Amadelli, R.
Amant, Carole
Amaretti, Alberto
Amarowicz, Ryszard
Amat, Anna
Amo-Ochoa, Pilar
Amorati, Riccardo
Amoroso, Rosa
AMOURI, Hani
An, Hyo-Jin
Anas, Andrea Roxanne
Andersen, Birgitte
Anderson, Alan
Anderson, James C.
Anderson, Laura L.
Andrade, Leandro
Andrade, Paula
Andre, C.
Andrei, Graciela
Andreoli, Mirko
Andreoli, Roberta
Andrés, José-Ignacio
Andruszkiewicz, Ryszard
Anfossi, Laura
Ang, Ching Seng
Angela, Bisio
Angeles-Boza, Alfredo M.
Angelovich, Tom
Annabi, Borhane
Annibalini, Giosuè
Anraku, Makoto
Ansorena, Diana
Ansquer, Jean Claude
Antipina, Maria N.
Antoni, Gunnar
Antoniotti, Sylvain
Apone, Fabio
Appendino, Giovanni
Aprea, Eugenio
Aragoncillo, Cristina
Arai, Masayoshi
Aranda, Agustín
Arapitsas, Panagiotis
Aratani, Naoki
Arbelaiz, Aitor
Arcanjo, Daniel Dias Rufino
Arce, Lourdes
Ardisson, Janick
Argyropoulou, Aikaterini
Arias-Pérez, María-Selma
Ariga, Hiroyoshi
Ariga, Katsuhiko
Arihara, Keizo
Arimondo, Paola B.
Arion, Vladimir
Arkin, M.R.
Armstead, William M.
Arnason, John
Arnason, John T.
Arnesano, Fabio
arnhold, juergen
Arnold, Eva
Arnusch, Christopher
Arranz, Elena
Arterburn, Jeffrey B.
Arvapally, Ravi Kumar
Arya, Dev
Asai, Akira
Asai, Teigo
Asakawa, Tomohiro
Asakawa, Yoshinori
Asard, Han
Asensio, Gregorio
Asenstorfer, Robert E.
Ashida, Hitoshi
Asiago, Vincent M.
Ata, Athar
Atanasov, Atanas
Atef, Eman
Atluri, Venkata S.R.
Austarheim, Ingvild
Avallone, Rossella
Avarvari, Narcis
Avato, Pinarosa
Averbeck, Dietrich
Azuma, Akifumi
Azuma, Kazuo
Azuma, Toshifumi
Babu, Jegdish
Bach, Horacio
Bach, Peter
Bachawala, Praveen
Bácskay, Ildikó
Baczek, Tomasz
Badawi, Michael
Baek, Kwang-Hyun
Baek, Nam-In
Baggiani, Claudio
Baiceanu, Elisabeta
Baidakov, V.G.
Baik, Sang-Ho
Bailo, Bibiana Garcia
Baindur-Hudson, Swati
Bajerska, Joanna
Bakare, Oladapo
Baker, Paul
Balaban, Teodor
Balcar, Hynek
Baldursdottir, Stefania
Ball, Zachary T.
Baltazar, Fátima
Baltora-Rosset, Sylvie
Balzarini, Jan
Bana, Emilie
Bandiera, Tiziano
Banerjee, Sangeeta Ray
Bang, Jeong Kyu
Bannister, Tom
Banskota, Arjun H.
Bansode, Rishipal R.
Banwell, Martin G.
Bao, Lei
Baraldi, Pier Giovanni
Baran, Timothy M.
Baranac-Stojanovi, Marija
Barba, Francisco J.
Barbayianni, Efrosini
Barberá, Joaquín
Barbier, Olivier
Barbieri, Paola
Barbosa, André L.R.
Bardet, Michel
Barea, Carlos
Barlow, James W.
Baroni, María V.
Barraja, Paola
Barreira, João
Barreira, Luísa
Barrio, Pablo
Barrow, Russell
Barth, Stephan W.
Bartholomä, Mark D.
Basak, Saroj K.
Basha, Riyaz
Basini, Giuseppina
Basiricò, ssa Loredana
Bastani, Nasser
Bastian, Susan E.P.
Batchelor, Hannah K.
Batchelor, Warren
Battino, Maurizio
Baudequin, Christine
Bauer, Tomasz
Bautista-Gallego, J.
Bautman, Ivan
Bavaresco, Luigi
Bayindir, Zerrin Sezgin
Bayse, Craig A.
Bazylak, Grzegorz
Bazylińska, Urszula
Beal, Peter A.
Beale, David J.
Beauchemin, André M.
Beberok, Artur
Becerra, Judith X.
Becerra, Valentina
Beckmann, Manfred
Bednarczyk-Cwynar, Barbara
Bednarski, Patrick
Beelman, Robert
Beerhues, Ludger
Begley, David J
Bella, Federico
Bellés, José María
Bellis, Susan
Bello, Nicholas T.
Belmar, Julio
Belo, Isabel
Belton, P.S.
Benaglia, Maurizio
Benavides, Jorge Alejandro
Bendall, Linda J.
Benelli, Giovanni
Bengtsson, Gunnar B.
Béni, Szabolcs
Benitez, Juan Gomez
Benkő, Zoltán
Bennett, Eric P.
Benoit, Isabelle
Berdis, Anthony J.
Berger, Gilles
Bergström, Christel A.S.
Berhow, Mark
Berké, Benedicte
Berlicki, Łukasz
Bernal, Juana M.
Bernardi, Luca
Bertelsen, Ragna
Bertolasi, Valerio
Bertrand, Daniel
Bertucci, Carlo
Besada, Cristina
Bescond, Jocelyn
Besson, Thierry
Best, Michael D.
Betancor, M.B.
Bevilacqua, Antonio
Beyeh, N. Kodiah
Bhagavathy, Ganga Viswanathan
Bhat, Shridhar
Bhatia, Harpreet
Bhushan, Alok
Bhutoria, Savita
Biagini, Giuseppe
Bian, Zhao-Xiang
Bidwell, Gene L.
Biely, P.
Bierenstiel, Matthias
Biesaga, Magdalena
Billard, Isabelle
Biot, Christophe
Birch, Edward
Bishop, Anthony C.
Bishop, Gregory W.
Bishop, Özlem T
Bisti, Silvia
Bizzarri, Mariano
Bjørklund, Geir
Blade Segara, Maria Cinta
Blanc, Aurélien
Blanca, Arango-Gonzalez
Błaszczyk, Jarosław
Blay, Gonzalo
Bleve, Gianluca
Boag, Peter R.
Boehm, Volker
Boeriu, Carmen G.
Bogdanov, Milen G.
Bohlmann, Jörg
Bojarsko, Andrzej
Bolm, Carsten
Bolognesi, M Laura
Bonesi, Sergio M.
Bonikowski, Radosław
Bonnefond, Audrey
Bonnet, Pascal
Bonnet, Sylvestre
Bonsignore, Riccardo
Bonvin, Alexandre M.J.J.
Boo, Yong Chool
Boost, Maureen V.
Bora, Ram Prasad
Borges, Anabela
Borowska, Marta
Borrego, Eli
Borrelli, Francesca
Borska, Sylwia
Borthaku, Dulal
Bosch, Christine
Bosch, Jürgen
Boschi, Stefano
Boss, Paul K.
Botham, Kathy
Böttcher, Thomas
Bou-Ali, M. Mounir
Bouckaert, Julie
Bougis, Pierre
Bouquillon, Sandrine
Bourgault, Steve
Bourlaye, Fofana
Boussettaa, N.
Boutin, Jean
Bouziotis, Penelope
Bowden, Bruce F.
Boyd, Robert S.
Boydston, Andrew J.
Boyer, Gerard
Boyom, Fabrice Fekam
Boysen, Reinhard
Brachet, Etienne
Braeuer, Andreas
Branca, Ferdinando
Brand, D. Jaco
Brandão, Maria Das Graças
Brandt, Peter
Braslavsky, Silvia E.
Bratsos, Ioannis
Braun, Klaus
Braune, Annett
Bravo López, Susana B.
Brazauskas, Gintaras
Brecker, Lothar
Breckwoldt, Michael
Breder, Alexander
Breitenfeld Granadeiro, Luiza
Bremner, John B.
Bren, Urban
Bressy, Cyril
Breuss, Johannes
Brian Chia, C. S.
Brill, Florian H.H.
Brockhausen, Inka
Broekhuis, A.A.
Bromann, Kirsi
Bronze, Maria
Brooks, Allen
Bros, Matthias
Brown, Mark A.
Brown, Peter J.
Bruchhaus, Iris
Brudzynski, Katrina
Bruhn, Torsten
Bruin, Bas De
Brullo, Chiara
Brun, Reto
Brunetti, Cecilia
Bruni, Giovanna
Bruno, Richard S.
Brustovetsky, Nickolay
Bryant-Friedrich, Amanda C.
Brycki, Bogumił
Brzozowski, Tomasz
Buchet, Rene
Bucio, José López
Buckley, Kevin
Budzisz, Elzbieta
Buechler, Christa
Bufo, Sabino A.
Bugarin, Alejandro
Bugg, Timothy D.H.
Bui, Bernadette Tse Sum
Bullon, Pedro
Burgos-Hernandez, Armando
Burkett, Brendan A.
Burnett, John C.
Buron, Frédéric
Burrows, Hugh
Buzzini, Pietro
Cabeza, Aurelio
Cabral, Jaydee
Cabrera, Carmen
Cabrera, Margarita Gutiérrez
Cadahía, Estrella
Caffrey, Patrick
Cafiero, Mauricio
Cai, Lisheng
Cai, Shuowei
Cai, Wenqing
Cai, Zhengxin
Calani, Luca
Calderon, Antonio
Calderon, Pedro Buc
Calderone, Vincenzo
Calero, Nuria
Calhelha, Ricardo C.
Callahan, Laura Smith
Camaioni, Emidio
Caminade, Anne-marie
Campagna, Shawn R.
Campanella, Beatrice
Canals, Daniel
Canas, Sara
Canesi, Sylvain
Canini, Antonella
Cannon, Richard D.
Cansby, Emmelie
Cantillo, David
Cañuelo, Ana
Cao, Bo
Cao, Song
Capasso, Raffaele
Capece, Angela
Caporaso, Nicola
Capperucci, Antonella
Caraglia, Michele
Carballeira, Nestor M.
Carbone, Anna
Carbonell Barachina, Angel A.
Carbonell-Bejerano, Pablo
Carcelli, Mauro
Cardoso, Susana M.
Carimi, Francesco
Carlberg, Carsten
Carlmark, Anna
Carlomagno, Teresa
Carlyle, James
Carmeli, Shmuel
Carosio, Federico
Carradori, Simone
Carranza, Maria G.
Carrau, Francisco M.
Carrico-Moniz, Dora
Carro-Díaz, A.M.
Carrubba, Alessandra
Carta, Antonio
Carta, Fabrizio
Carulla, Natàlia
Carvajal, M.
Carvalho Jr., Luiz Bezerra
Casamenti, Fiorella
Casassa, L. Federico
Cascioferro, Stella
Casettari, Luca
Castaneda, Homero
Castell, Alina
Castellari, Massimo
Castilho, Paula C.
Castro, Angeles
Castro, Antonio Jesus
Casuscelli, Sandra G.
Catauro, Michelina
Catto, Marco
Cavaco-Paulo, Artur
Cavaleiro, C.
Cavalli, Roberta
Celia, Christian
Cena, Hellas
Cerecetto, Hugo
Cerella, Claudia
Cermak, R.
Cerqueira, Nuno Sousa
Ceruso, Mariangela
Céspedes, Carlos L.
Cha, Hyukjin
Chacón, Iván De-La-Cruz
Chagnes, Alexandre
Chai, Han-Ha
Chai, Yifeng
Chaleix, Vincent
Chambin, O.
Chan, Chin-feng
Chan, Thomas
Chan, Wan
Chan, Yu-Yi
Chang, Chih-Jui
Chang, Fang-Rong
Chang, Hsueh-Wei
Chang, Il-moo
Chang, Kyung-Soo
Chang, Sue-Joan
Chang, Tsu-Chung
Chang, Tsung-Hsien
Chang, Yuanshiun
Chappell, Joseph
Chaput, John C.
Chapyshev, Sergei V.
Chatel, Grégory
Chatfield, David
Chatterjee, Jhinuk
Chatterjee, Parnali
Chaud, Marco V.
Chauhan, Harsh
Chavez, Ferman
Chelli, Riccardo
Chemat, Farid
Chen, Bo
Chen, Chao
Chen, Cheng-Lung
Chen, Chi
Chen, Chung-Yi
Chen, Chunjung
Chen, Xiaozhuo
Chen, Gen-Hung
Chen, Hsin-Chun
Chen, Hsuan-Ying
Chen, Huaiqiong
Chen, Jih-Jung
Chen, Kew-Yu
Chen, Li
Chen, Liao Y.
Chen, Lingxin
Chen, Long-fang Oliver
Chen, Miaw-Ling
Chen, Pei
Chen, Qiaohong
Chen, Qi-he
Chen, Ruei-Tang
Chen, Shangwu
Chen, Shaowei
Chen, Shuai
Chen, Shuqing
Chen, Tianbao
Chen, Xiao-Yin
Chen, Xiuping
Chen, Yann-Jang
Chen, Yen-Ling
Chen, Ying-Chun
Chen, Yung-Hsiang
Chen, Yun-Wen
Chen, Zhilei
Chen, Zili
Cheng, Juei-Tang
Cheng, Si-Xue
Chern, Ji-Wang
Chern, Ming-Kai
Chernikov, Oleg
Cheung, Randy Chi Fai
Chevet, Eric
Chew, Eng-Hui
Chi, Lianli
Chianese, Anthony
Chiang, Hsiu-Mei
Chiang, Po-Chang
Chiang, Tai-an
Chiang, Wen-Dee
Chida, Noritaka
Chien, Tun-Cheng
Chilin, Adriana
Chin, Michael
Chin, Young-Won
Chiosis, Gabriela
Chiou, Antonia
Chiou, Chun-Tang
Chiou, Hui-ling
Chiou, Wen-fei
Chitwood, David
Chiu, Chien-Chih
Chiu, Gigi N.C.
Chiu, Kuo-Ping
Chiu, Tai-chia
Chiva-Blanch, Gemma
Chmielewski, Marek
Cho, Cheong-Weon
Cho, Hyunah
Cho, Man-Ho
Cho, Somi Kim
Cho, Sung-Dae
Cho, Won-Kyung
Chobot, Vladimir
Choi, Hyeok
Choi, Hyoung Jin
Choi, Hyukjae
Choi, Hyung-Kyoon
Choi, In-Geol
Choi, Inho
Choi, Jae-Hong
Choi, Yong Suk
Choi, Young Whan
Choi, Yung Hyun
Chong, Youhoon
Chorianopoulos, Nikos G.
Chou, Kan-sen
Chou, Kuo
Chou, Stanley
Chou, Tsui-Fen
Chou, Wing-ming
Christ, Frauke
Christensen, Jørn B.
Christensen, Kathrine B.
Chu, Shidong
Chu, Tin-Chun
Chua, Lee Suan
Chuang, Jing-Jing
Chuang, John Shih-Ching
Chuang, Ta-hsien
Chuburu, Françoise
Chudasama, Vijay
Chueh, Pin Ju
Chwalibog, André
Chyau, Charng-Cherng
Cibulka, Radek
Cicero, Arrigo F.G.
Cichero, Elena
Cidade, Honorina
Cimpoiu, Claudia
Ciochina, Roxana
Cirillo, Giuseppe
Cirone, M.
Cirrincione, Girolamo
Cisnetti, Federico
Citterio, Barbara
Clark, Daniel A.
Clark, Ewan R.
Clark, Lindsay V.
Clark, Robert D.
Clark, Timothy
Clarke, Anthony
Claudio, Ana Filipa
Claus, Harald
Clausen, Morten Rahr
Clement, Bernd
Clément, Sébastien
Clementi, Emilio
Clements, Carol
Cliff, Margaret
Clyburne, Jason
Cobbold, Simon
Coccetti, Paola
Coelho, Jaime
Coelho, José A
Coelho, Paulo
Cognasse, Fabrice
Coiffard, Laurence J.M.
Coll, Josep
Collina, Simona
Collins, J. Grant
Collins, Scott D.
Colombo, Giancarlo
Colombo, Maria Laura
Colotti, Gianni
Colquhoun, Thomas A.
Compagnone, Dario
Conceição, Katia
Conklin, Daniel
Consonni, Roberto
Conti, Barbara
Contreras-Castillo, Carmen J.
Cook, Gregory M.
Copani, Agata
Copolovici, Dana Maria
Cordero, Mario
Cordewener, Jan H.G.
Cordoba-Diaz, D.
Coronas, Joaquín
Correia, Jack
Cortajarena, Aitziber L.
Cortes, Andres J.
Corvis, Yohann
Costa, Elson Alves
Costa, Paulo J.
Costa, Pedro Reis
Costantino, Valeria
Costi, Roberta
Cotten, Myriam L.
Couce, María D.
Coutinho, João Paulo
Couture, Manon
Cowan, James
Cowland Jack, Jack B.
Cragg, Peter
Crean, Daniel
Creaven, Bernie
Crespan, Emmanuele
Crestini, Claudia
Cretich, Marina
Crispin, Matthew C.
Crisponi, Guido
Crowley, Peter B.
Csallany, A. Saari
Csámpai, Antal
Csuk, René
Csupor, Dezső
Cubry, Philippe
Cudic, Predrag
Cueto, Mercedes
Cui, Jian-Zhong
Cui, Mengchao
Cunha, Anna C.
Currais, Antonio
Curti, Claudio
Curtis, Jonathan M.
Custódio, Luísa
Cutignano, Adele
Cycoń, Mariusz
CZAJKOWSKI, Robert
Czyczyło-Mysza, Ilona
D’Acquarica, Ilaria
D’Agostino, Martin
D’Alessio, Cecilia
Da Ros, Tatiana
D'abrosca, Brigida
Dagnino, Lina
Dagorne, Samuel
Dai, Wei-Lin
Dai, Yumin
Dai, Zhanwu
Daiber, Andreas
Dalko, Peter I.
D'all aqua, Stefano
Dall’Olio, Fabio
Dallinger, Doris
D'Ambrosio, Michele
Dammne, Els Van
Danaher, Martin
Dang, Jing-Shuang
Darder, Margarita
Darvin, Maxim
Darwiche, Nadine
Dasgupta, Sayani
D’Auria, Maria Valeria
David Wang, Hui-Min
David, Jorge
David-Cordonnier, Marie-hélène
Davidson, Mark
Davies, Christopher
Davies, Kay E.
Davies, Stephen G.
Day, Brian J.
De Almeida Coelho De Sousa, Maria João
De Alvarenga, Elson Santiago
De Barros, Dragana
De Beer, Dalene
De Cienfuegos, Luis Álvarez
De Falco, Enrica
De Feo, Vincenzo
De Gier, Jan-Willem
De La Fuente - Núñez, César
De la Fuente, Helena
De la Hoz, Antonio
De La Mata, Javier
De Lera, Ángel R.
De Lucena, J.M.V.M.
De Martino, Laura
De Oliveira Otto, Marcia C.
De Paula, Leonardo
De Paz, Jose Luis
De Rivero Vaccari, Juan Pablo
De Visser, S.P.
De Witte, Peter A.M.
Dea-Ayuela, María Auxiliadora
Dean, Lisa
Debbert, Stefan L.
Debyser, Zeger
DeCarlo, Arthur A.
Decristoforo, Clemens
Deev, Sergey L.
Deglmann, Peter
Deharo, Eric
Dekanski, Dragana
Del Campo, Teresa Martínez
Del Castillo, M.L.R.
Del Hierro, Isabel
Del Pozo Losada, Carlos
Delattre, Cédric
Delattre, François
Delcamp, Jared H.
Delgado, Angel
Delgado, Rita
DeLorenze, Gerald N.
Delsuc, Nicolas
Deluc, Laurent G.
Demain, Arnold L.
Demeunynck, Martine
Demopoulos, Vassilis J.
Demuez, Marie
Dénes, Szieberth
Deng, Yinyue
Deng, Yongming
Dennis, Jonathan J.
Desai, Umesh R.
Deshpande, Deepak A.
Desmet, Tom
Dessen, Andréa
Detsi, Anastasia
Detty, Michael R.
DeWitt, Jamie C.
Dhau, Jaspreet S.
Di Pierro, Prospero
Diana, Patrizia
Diana, Patrizia
Díaz-Ortiz, Ángel
Dickert, Franz L.
Dietzek, Benjamin
Díez, David
Diksic, Mirko
Dilman, Alexander D.
Dimitroff, Charles J.
Dimmock, Jonathan R.
Ding, Chunbang
Ding, Xiaobin
Disney, Matthew D.
Diwald, Oliver
Djiabourov, Madeleine
Djordjevic, Steven P.
Dolzhenko, Anton V.
Doménech-Carbó, Antonio
Domenico, Schillaci
Domingo, Luis R.
Domínguez Álvarez, Enrique
Domizio, Paola
Donald, Kelling J.
Donkor, Kingsley K.
Donzelli, Sonia
Donzello, Maria Pia
Dosio, Franco
Dossett, Michael
Douroumis, Dionysios
Dovizio, Melania
Downey, Gerard
Drabowicz, Jozef
Dracinsky, Martin
Dragišić Maksimović, Jelena J.
DRAYE, Micheline
Drickamer, Kurt
Drulis-Kawa, Zuzanna
du, zhenting
Duan, Wei
Duan, Yourong
Ducheyne, Paul
Dudding, Travis
Dudek, Marta K.
Dudley, E.D.
Dudukovic, Milorad P.
Dudziak, Diana
Dughera, Stefano
Duh, Chang-Yih
Duke, Colin
Duke, Stephen O.
Dumont, Matthieu
Duncan, James
Dunietz, Barry D.
Dunn, Robert O.
Durán, Nelson
Durán-Patrón, Rosa
Durazzo, Alessandra
D'Urso, Alessandro
Dusane, Devendra
Dutkowski, Greg
Dutot, Mélody
Dzantiev, Boris
Dziedzic, Arkadiusz
Easmon, Johnny
Ebbinghaus, Simon
Eder, Matthias
Edison, Arthur
Edmondson, Dale
Edwards, Everard
Edwards, Katie
Eeckhaut, Ann Van
Efimia, Hatzidimitriou
Ehlting, Jürgen
Ehrhardt, Carsten
Eitsuka, Takahiro
Ekuni, Daisuke
El Kaim, Laurent
Elez-Martinez, Pedro
El-Ghayoury, Abdelkrim
Elhardami, Abdelbasset
Elleuche, Skander
El-Seedi, Hesham R.
Elsinghorst, Paul W.
El-Soda, Mohamed
Emmerich, Michael
Emslie, David
Endo, Akihito
Engel, Nadja
Engels, Joachim
Engman, Lars
Enguita, Francisco
Enríquez, Raul G.
Enthaler, Stephan
Eparvier, Veronique
Epstein, Irwing
Erdélyi, Máté
Eritja, Ramon
Ertl, Peter
Erxleben, Andrea
Escudero-Gilete, M. Luisa
Escuredo, Olga
Espinosa-Aguirre, Jesús Javier
Esposito, Emilio Xavier
Esposito, Sergio
Esteban Cerdán, Luis
Esteban, Maria Angeles
Esteruelas, Miguel A.
Esteve-Romero, Josep
Estévez, Francisco
Estévez-Braun, Ana
Estevinho, Leticia
Estrela, José M.
Estrine, Boris
Euaggelos, Gikas
Evangelou, Michael Wassilios
Evanno, Laurent
Evans, Barbara R.
Evans, Jason
Evans, Paul
Ewald, Collin
Exarchou, Vassiliki
Faber, Kurt
Fabiano-Tixier, Anne-Sylvie
Fabien, Robert Peillard
Fahmy, Hesham
Fairlamb, Ian
Faita, Giuseppe
Fan, Wei
Fang, Bai
FANG, Evandro Fei
Fang, Jianguo
Fang, Wenwen
Fanizzi, Francesco
Fanning, Kent J.
Faraldos, Juan A.
Farce, Amaury
Farnham, Mark W.
Faure, Sébastien
Faustino, Maria Amparo
Favier, Isabelle
Favre-Réguillon, Alain
Fawzi, Lux
Feás, Xesús
Felpin, François-xavier
Felsner, M.L.
Feng, Shou Hua
Feng, Yangbo
Feng, Yibin
Feng, Yu
Fennell, Timothy R.
Fenner, Amanda
Fernandes, Carla
Fernandes, Patricia
Fernandes-Ferreira, Manuel
Fernandes-Pedrosa, Matheus de F.
Fernandez Lucas, Jesus
Fernández, José J.
Fernandez, Pedro Alexandrio
Fernandez-Lafuente, Roberto
Fernández-Lázaro, Fernando
Ferrari, Erika
Ferrari, Stefania
Ferraris, Ronaldo P.
Ferraz, Ricardo
Ferreira Mendes, Alexandrina
Ferreira, Arthur S
Ferreira, Sandra Regina Salvador
Ferrer Montiel, Antonio
Ferretti, Valeria
Ferrins, Lori
Fessard, Thomas C.
Fiedor, Leszek
Figadere, Bruno
Figueiredo, Ana Cristina
Figueiredo, Isabel Vitória
Figueiredo, Marxa L.
Filarowski, Aleksander
Filgueira, Carly
Filho, Benedito Prado Dias
Filippetti, Ilaria
Fiocco, Daniela
Fiorini, Dennis
Fiorucci, Stefano
Fischer, Wolfgang B.
Fischmeister, Cedric
Fisher, Jed F.
Fitches, Elaine C
Fitzenberger, Elena
Flamini, Guido
Flemmig, Jörg
Fletcher, Mary
Flint, Harry J.
Floreancig, Paul E.
Flox, Cristina
Fochi, Mariafrancesca
Fontaine, Fred
Fonteh, Alfred N.
Forbey, Jennifer S.
Forgan, Ross S.
Formisano, Carmen
Fossa, Paola
Foster, E. Johan
Fosu-Mensah, Nelly
Fotakis, Charalambos
Foubelo, Francisco
Fox, Keith R.
Franc, Benjamin
Franchi, Nicola
Francis, Leigh
Franciscis, Vittorio De
Franck, Xavier
Frantz, Doug
Freccero, Mauro
Frega, Natale
Freigang, Stefan
Freund, Nadja
Friberg, Magne
Fribley, Andrew M.
Frija, Luís M.T.
Froeyen, Matheus
Fröhlich, Eleonore
Frolova, Lilya
Frontera, Patrizia
Frontera, Antonio
Frotscher, Martin
Frutos, Maria Jose
Fu, Hua
Fu, Junjie
Fu, Liwu
Fu, Qiang
Fu, Zongming
Fujimori, Ko
Fujita, Masayuki
Fujita, Mikako
Fujita, Morifumi
Fujiwara, Kenshu
Fukaya, Takayuki
Fukuta, Shiro
Fukuzawa, Shin-ichi
Fülöp, Ferenc
Fülöp, Zoltán
Furukawa, Koichi
Fuselli, Fabio
G. Lago, João Henrique
Gaan, Sabyasachi
Gabius, Hans-Joachim
Gai, Francesco
Galán-Mascarós, José Ramón
Galan-Vidal, Carlos Andres
Galdiero, Massimiliano
Galeano-Díaz, Teresa
Galeazzi, Roberta
Gales, Luís
Galiano, Silvia
Gall, Thierry Le
Gallagher, John F.
Gallardo, Hugo
Galvão, Adelino M.
Galvez, Eva M.
Gambuti, A.
Gandin, Valentina
Ganguly, Bishwajit
Ganji, Purna Chandra
Gantenbein-Ritter, Benjamin
Gao, Hao
Gao, Jinming
Gao, Kun
Gao, Wen-yuan
Gao, Yuxing
Garattini, Enrico
Garcia Estévez, Ignacio
Garcia Rojas, Edwin E.
Garcia, Iñaki
García, Jeannette M.
García-Álvarez, Joaquín
García-Álvarez-Coque, María C.
Garcia-Bennett, Alfonso E.
Garcia-Granda, Santiago
García-Martínez, Santiago
Garcia-Vallve, Santiago
García-Valverde, María
Garde-Cerdán, Teresa
Gardner, Dale
Garneau-Tsodikova, Sylvie
Garrido, Daniel Valero
Garrigues Pelufo, Teresa
Garrison, Thomas F.
Garson, Mary
Gaspar, Diana
Gasparrini, Massimiliano
Gasparutto, Didier
Gasser, Christoph
Gates, Derek P.
Gaumann, Andreas
Gautier, Arnaud
Gawlik- Dziki, Urszula
Gazit, Ehud
Ge, Qinyu
Geary, Sean
Geary, Timothy G.
Geiger, Jonathan D.
Gein, V.L.
Genta-Jouve, Grégory
Gentile, Carla
Gentz, Margaret
Georgakilas, Alexandros G.
Georgi, Anett
Georgiev, Milen
Georgiev, Vasil
Geronikaki, Athina
Gerothanassis, Ioannis P.
Ghafoor, Abdul
Ghanotakis, Demetrios F.
Giacosa, Simone
Giampiei, Francesca
Giancane, Gabriele
Giannini, Giuseppe
Giaouris, Efstathios
Gibbons, Simon
Gijzen, Mark
Gil, Maria H.
Gilard, Véronique
Gill, Chris Ir
Gille, Lars
Gillies, Graeme
Gillinham, Dennis
Gil-Muñoz, Rocío
Gimenez, J.
Gimeno, M. Concepción
Gimeno, Miquel
Giraudeau, Patrick
Girbes, Tomás
Girgih, Abraham T.
Gironés-Vilaplana, Amadeo
Giuliani, Francesco
Giuliano, Robert M.
Gizdavic-Nikolaidis, Marija R.
Glauser, Gaetan
Gleiser, Raquel M.
Gliszczyńska, Anna
Głuszyńska, Agata
Gniewosz, Małgorzata
Gobis, Katarzyna
Godino-Salido, Maria Luz
Goel, Ramesh
Gofferjé, Gabriele
Goławska, Sylwia
Gold, Ben
Goldberg, Alfred
Golding, Jon P.
Goldman, Alan
Golovko, Mikhail
Gomes, Andreia
Gomes, Sónia
Gómez Di Marco, Perla
Gómez-Sandoval, Zeferino
Gonçalves, M. Sameiro T.
Gonda, Sándor
Gong, Xingchu
Gonthier, Marie-paule
Gontier, Eric
González San-José, María Luisa
Gonzalez, Antonio
González-Coloma, Azucena
González-Díaz, Humberto
González-Gómez, José C.
González-Marrero, Joaquin
González-Mas, M. Carmen
González-Pacanowska, Dolores
González-Stuart, Armando
Gorinstein, Shela
Gorlach, Sylwia
Gorman, Christopher B.
Gorman, Gregory S.
Gorrell, M.D.
Goslinski, Tomasz
Gosmann, Grace
Gosselet, Fabien
Gosselin, Gilles
Goto-Yamamoto, N.
Gou, Shao-Hua
Gouault, Nicolas
Goulas, Vlasios
Gourrier, Aurelien
Goya, Luis
Graaf, Rob M. de
Grab, Dennis
Grabowski, Slawomir
Grabulosa, Arnald
Graham, Ann
Graier, Wolfgang
Graikou, Dia
Grainger, Richard
Granado, M. Dolores
Grandjean, Agnès
Granica, Sebastian
Grant, Irene
Grassi, Giovanni
Gray, Christopher A.
Gray, Sandra
Grayer, Renée J.
Grazioso, Giovanni
Greco, Antonio
Greco, Claudio
Green, Christopher
Greenberg, Robert M.
Greenwood, Michael T.
Grevy, Raquel
Grider, Arthur
Grierson, David
Grogan, Gideon
Groll, Michael
Groot, Marie Louise
Grosu, Ion
Grovel, Olivier
Grover, Ashok K.
Gruselle, Michel
Gruszecki, Wieslaw
Grynkiewicz, Grzegorz
Grzebelus, Dariusz
Gu, Hongwei
Gu, Jingkai
Gu, Jinlou
Gu, Junfeng
Gualandi, Andrea
Gudiña, Eduardo
Guglielmo, Stefano
Guido, Luis
Guilhon, Giselle Maria S.P.
Guillena, Gabriela
Guindon, Josee
Guiné, Raquel P.F.
Gulino, Antonino
Gumienna-Kontecka, Elżbieta
Gunia-Krzyżak, Agnieszka
Guo, Fenghai
Guo, Hongchao
GUO, PU
Guo, Shouwu
Guo, Ya
Guo-Qing, Zhang
Gupton, B. Frank
Gurney, Richard
Gustafson, Kirk
Gütschow, Michael
Guvench, Olgun
Guzik, Urszula
Guzman, Monica
Habaue, Shigeki
Hadden, Kyle
Hadjikakou, Sotiris K.
Hadjipavlou-Litina, Dimitra
Haglund, Caj
Haiges, Ralf
Haino, Takeharu
Hak, Sjoerd
Ham, Jong Hyun
Hamad-Scifferli, Kimberly
Hamaguchi, Masahide
Hamasaki, Katsuyuki
Hameed, Shahid
Hammer, Kathrine
Hammes, Ulrich Z.
Han, Hogyu
Han, Jianlin
Han, Quanbin
Han, Rui-Min
Han, Se Jong
Hanks, Timothy
Hanley, Quentin S.
Hano, Christophe
Hansen, Henrik C.
Hanson, Robert N.
Hao, Dacheng
Hao, Hongdong
Haouas, Mohamed
Hara, Shoji
Hara, Yusuke
Harbertson, James F.
Harduin-Lepers, Anne
Harper, Jason B.
Harris, Cory S.
Harris, Lynne
Harris, Philip
Harrison, William T.A.
Hartman, Matthew
Hasegawa, Morifumi
Hashimoto, Makoto
Hashimoto, Masaru
Hashimoto, Takashi
Hashmi, A. Stephen K.
Hatae, Noriyuki
Hatano, Tsutomu
Haufe, Günter
Häusler, Rainer E.
Havill, Nathan
Havlík, Jaroslav
Hayes, Sophia C.
He, Chengwei
He, Di
He, Jing
He, Lang-Chong
He, Li-Min
He, Qingfang
He, Zhengjie
Hearn, Michael J.
Hearn, Milton
Hebert, Daniel N.
Heckman, Charles J.
Heimler, Daniela
Heinrichs, David
Helaly, Soleiman
Helm, Richard
Helmke, Elisabeth
Helmut Martin, Hügel
Henderson, Luke
Hennebelle, Thierry
Hennig, Andreas
Hennig, Ewa
Henrich, Curtis J.
Henriques, Maria G
Henriques, Marta H.F.
Henshall, David C.
Hent, Andrei
Heras, Angeles
Herdewijn, Piet
Hermans, Ive
Hermosin, Isidro
Hernádi, Klára
Hernández, Francisca
Hernández, Pablo E.
Herrera, Raquel P.
Herrero, Laura
Herrmann, Hartmut
Heutz, Sandrine
Hewitson, Peter
Hickey, Anthony J.
Hideg, Kálmán
Hideyuki, Mitomo
Highfield, James George
Hihara, Yukako
Hikawa, Hidemasa
Hiller, Karsten
Himo, Fahmi
Hirabayashi, Jun
Hirai, Itaru
Hirakawa, Hidehiko
Hirano, Tomoya
Hirasawa, Noriyasu
Hirashima, Akinori
Hirota, Takashi
Hladky, Stephen B.
Ho, Su-Chen
Ho, Yi-Cheng
Hocek, Michal
Hocková, Dana
Hodge, Philip
Hofer, Tim
Hofmann, Alan F.
Hofmann, Bettina
Hofmann, Ute
Hogg, Philip
Hollenstein, Marcel
Holliday, John
Holm, René
Holst, Otto
Holzer, Wolfgang
Honda, Tadashi
Hondal, Robert
Hong, Chin-Yih
Hong, Mee Young
Hong, Yong-Han
Hook, Ingrid L.I.
Horbowicz, Marcin
Horino, Yoshikazu
Horvath, Dragos
Hoskins, Clare
Hosokawa, Seijiro
Hosoya, Takamitsu
Hostetler, Karl
Hou, Chien-Wei
Hou, Harvey
Hou, Ming-feng
Hou, Ming-Hon
Houen, Gunnar
Houng, Jer-yiing
Hovorun, Dmytro M.
Hoyos, Pilar
Hrincius, Eike
Hsia, Shih-Min
Hsiao, George
Hsieh, Jung-Feng
Hsieh, Pei-wen
Hsieh, Tusty-Jiuan
Hsieh, Yih-Shou
Hsing, Chung-Hsi
Hsu, Chin-lin
Hsu, Hsiu-Fu
Hsu, Tc
Hu, Deyu
Hu, Jiang
Hu, Jinbo
Hu, Miao-Lin
Hu, Ping
Hu, Qiao-Sheng
Hu, Qingzhong
Hu, Xiang-Ping
Hua, Huiming
Hua, Ruimao
Hua, Yufei
Huang, A-mei
Huang, Chi-Chang
Huang, Chih-Ching
Huang, Feihe
Huang, George Tzou-Chi
Huang, Hui-Chi
Huang, Li
Huang, Lin-Fang
Huang, Ling
Huang, Mingdong
Huang, Sheng-Teng
Huang, Sheng-You
Huang, Tien L.
Huang, Wei-Chien
Huang, Wei-Jan
Huang, Wenlin
Huang, Yadong
Hubka, Vit
Huczyński, Adam
Hudnall, Todd W.
Huefner, Antje
Huh, Jin Hoe
Huigens III, Robert W.
Hui-Ting, Chang
Hulme, Christopher
Hunyadi, Attila
Huq, Fazlul
Hureau, Christelle
Hussein, Ahmed A.
Hwang, Bang Yeon
Hwang, Dae Youn
Hwang, Gil Tae
Hwang, Ming-Jing
Hwu, Jih-Ru
Hynes, Russell K.
Iannazzo, Daniela
Ichikawa, Satoshi
Ichikawa, Takahiro
Iglesias, Marta
Ignat, Adriana Grozav
Ignea, Codruţa
Ihara, Eikichi
Iiyama, Kazuhiro
Ikebukuro, Kazunori
Illa, Ona
Inokuchi, Tsutomu
Inoue, Katsutoshi
Inoue, Shigeyoshi
Inoue, Tomoya
Iorizzi, Maria
Irakli, Maria
Isaacs, John T.
Isaka, Yoshitaka
Isaza Martínez, José Hipólito
Ishihara, Atsushi
Ishihara, Kazuyuki
Ishikura, Minoru
Islam, Md Soriful
Ismaili, Lhassane
Isman, M.B.
Isman, Murray B.
Isshiki, Yoshiaki
Ito, Shingo
Ito, Takuya
Ito, Yoichiro
Itoh, Toshiyuki
Ivanova, Bojidarka
Ivanovska, Nina
Iwaoka, Michio
Iwasaki, Tomohiro
Iwata, Tadahisa
Iwata, Yuko
J. Sinanoglou, Vassileia
Jacob, Claus
Jacobsen, Marianne C
Jacquot, Yves
Jaffrey, Samie R.
Jagerovic, Nadine
Jain, Prashant
Jaiswal, Rakesh
Jaki, Birgit
Jäkle, Frieder
Jalkanen, Aaro J.
Janczarek, Monika
Jang, Dae Sik
Jang, Hyeung-Jin
Jang, Ik-Soon
Jang, In-Cheol
Janin, Joël
Jankowski, Michael
Jansen, Eugen
Jansen, Rolf
Jara-Palacios, M. José
Jarmoluk, Andrzej
Jasinski, Jerry P.
Javadi, Ali
Jayant, Rahul Dev
Jaye, David L
Jazirehi, Ali
Jembrek, Maja Jazvinšćak
Jenks, William
Jenssen, Håvard
Jeong, In Howa
Jeong, Lak Shin
Jeong, Seon-Yong
Jeong, Tae-Sook
Jeong, Woo-Sik
Jerković, Igor
Jerz, Gerold
Jessel, Nadia
Jessen, Henning Jacob
Jewett, John
Jha, Mukund
Ji, Bin
Ji, Debin
Ji, Nai-Yun
Ji, Tengfei
Jia, Jianfeng
Jia, Zhe
Jianbo, Xiao
Jiang, Bo
Jiang, Mali
Jiang, Yong
Jiang, Yueming
Jiang, Yuyang
Jiang, Zhiyong
Jiang, Zi-Hua
Jimeno, Ciril
Jin, Jin
Jin, Tienan
Jindrich, Jindrich
Jing, Jing
Joaquin, Plumet
Johnson, John A.
Jois, Seetharama
Jones, Huw
Jones, Jessica L.
Jones, Meriel
Jordão, António M.
Jorge-Sobrido, Ana Belén
Jose, Pedro A
Joseph, Lucy
Jourdes, Michael
Jovanovic, Jelena D.
Joven, Jorge
Juang, Tzong-Yuan
Jubault, Philippe
Jun, Hee-Sook
Jung, Friedrich
Jung, Klaus
Jung, Sang Hun
Jurado, Amália S.
Jurga, Stefan
Juskowiak, Bernard
Justicia, José
Justino de Almeida, Celine
Juszczak, Lesław
Jyh-fei, Liao
Kabatcc, Janina
Kachko, Alla
Kachlicki, Piotr
Kaczor, Agnieszka
Kaddoumi, Amal
Kadokawa, Jun-ichi
Kadomatsu, Kenji
Kadota, Kazunori
Kafarski, Pawel
Kaiser, Markus
Kakizaki, Tomohiro
Kakkar, Ashok K.
Kakuchi, Ryohei
Kalantar-zadeha, Kourosh
Kalemos, Apostolos
Kalinowska-Lis, Urszula
Kaliora, Andriana C
Kallenbach, Neville
Kaltner, H.
Kamatou, Guy P.
Kamei, Toshiyuki
Kamenetsky, Rina
Kamiloglu, Senem
Kamiński, Zbigniew
Kamisuki, Shinji
Kamiya, Masakatsu
Kammerer, Robert
Kanai, Masashi
Kanan, Yogita
Kanaoka, Masahiro
Kaneco, Satoshi
Kaneo, Y.
Kang, Bum-Yong
Kang, Chulhun
Kang, Dun-Yen
Kang, Hee Eun
Kang, Hee-Wan
Kang, Jong Seong
Kang, Jonghoon
Kang, Myung Joo
Kang, S.C.
Kang, So Young
Kang, Sumin
Kang, Yun
Kanias, TAMIR
Kankanala, Jayakanth
Kannan, Srinivasaraghavan
Kantminienė, Kristina
Karakhanov, Edward
Karapanagiotis, Ionnis
Karim, Mohammad R.
Karuppagounder, Saravanan S.
Kaschula, Catherine H.
Kashina, Anna
Kashiwada, Yoshiki
Kashman, Yoel
Kasiotis, Konstantinos M.
Kasperczyk, Sławomir
Katagiri, Hiroshi
Kataoka, Hiromi
Katayama, Shigeru
Kato, Hikaru
Kato, Koichi
Katsila, Theodora
Kaufman, Teodoro S.
Kaufmann, Dieter
Kauppinen, Anu
Kaurinovic, Biljana
Kawabata, Kyuichi
Kawahara, Teppei
Kawai, Yoshichika
Kawakami, Susumu
Kawamura, Fumio
Kawiak, Anna
Kay, Graeme
Kayaki, Yoshihito
Kayano, Shin-ichi
Kayser, Oliver
Keefover-Ring, Ken
Keire, David A.
Kellogg, Josh
Kellow, NJ
Kelly, Charles
Kenny, Owen
Kenyon, William J.
Keong, KWOH Chee
Kesharwani, Tanay
Keski-Rahkonen, Pekka
Kessler, Vadim
Keul, Helmut
Keum, Gyochang
Keusgen, Michael
Khiari, Zied
Khlebnikov, Alexander F.
Khoury, Madona
Khrustalev, Viktor
Khun, Nay Win
Khursigara, Cezar M.
Kido-Nakahara, Makiko
Kiec-Kononowicz, Katarzyna
Kiefel, Milton
Kijjoa, Anake
Kikuchi, Haruhisa
Kilpeläinen, P.O.
Kim, Beom Seok
Kim, Choongik
Kim, Chul Young
Kim, Dong Hyun
Kim, Dongsup
Kim, Hak Yong
Kim, Ho Jin
Kim, Hocheol
Kim, Hye Kyong
Kim, Hyeung-Rak
Kim, Hyung Sik
Kim, Hyunwoo
Kim, Jeong Hun
Kim, Ji Yeon
Kim, Jin-Kyung
Kim, Jung-ae
Kim, Jungahn
Kim, Junghyun
Kim, Kyoung Hoon
Kim, Kyung-Min
Kim, Kyung-su
Kim, Mi-hyun
kim, minsoo
Kim, Myunghee
Kim, No Soo
Kim, S.J.
Kim, Sung-Hoon
Kim, Tae Kon
Kim, Yeong-Shik
Kim, Yeon-Ju
Kim, Young Woo
Kim, Young-Kyoon
Kim, Youngsoo
Kim, Yun-bae
Kim, Yuri
Kimura, Hideto
Kimura, Tsutomu
Kimura, Yoshiharu
Kimura, Yoshinobu
King, Frank D.
Kinoshita, Takayoshi
Kirchmair, Johannes
Kireev, Dmitri
Kirsch, Gilbert
Kiselev, K.V.
Kiss, Anna K.
Kiss, Robert
Kitagawa, Toshikazu
Kitagishi, Hiroaki
Kitamura, Shigeyuki
Kitamura, Tsugio
Kittaka, Atsushi
Kizaka-Kondoh, Shinae
Klapötke, Thomas
Klempier, Norbert
Klitgaard, Janne
Knall, Astrid-Caroline
Knappe, Daniel
Knight, David W.
Knolhoff, Ann M.
Knölker, Hans-Joachim
Knupp, Gerd
Ko, Chun-hay
Ko, Kam Ming
Kobayashi, Kazuhiro
Kobayashi, Shingo
Kobori, Akio
Kobori, Hikaru
Koch, Oliver
Kochi, Takuya
Kockmann, Norbert
Koga-Ito, Cristiane Yumi
Koharudin, Leonardus M.I.
Köhler, Jürgen
Kohno, Yasushi
Kohno, Yuki
Kojima, Akiko
Kojima, Chie
Kojo, Shosuke
Kokotos, Christoforos G.
Kokou, Fotini
Kolaczkowski, Marcin
Kolehmainen, Erkki
Kolhar, Poornima
Kollár, László
Komeyama, Kimihiro
Komiyama, Makoto
Komori, Kikuo
Konarev, Dmitri
Kondo, Shin-ichi
Kondo, Tadashi
Kong, Dexin
Kong, In-soo
Kong, Lingxue
Koole, Leo H.
Kornev, Alexandr
Kortner, Trond M.
Košir, Iztok Jože
Kosova, Klara
Kostomitsopoulos, Nikolaos G.
Kotseridis, Y.S.
Koufaki, Maria
Koulman, Albert
Koundouras, Stefanos
Kouretas, Dimitrios
Kourkoutas, Yiannis
Koutoulis, Anthony
Kovacs, Lajos
Kowalski, Konrad
Koziel, Jacek
Kožíšek, Jozef
Krag, Thomas O
Krajka-Kużnik, Violetta
Krasnikov, Boris
Krchňák, Viktor
Kreis, Wolfgang
Kriegsheim, Alex Von
Krishnakumar, Vivek
Krokida, Magdalini
Krol, Wojciech
Kruss, Sebastian
Krutovsky, Konstantin V.
Krystof, Vladimir
Ku, Kuo-Lung
Kuan, Yu-Hsiang
Kuberan, Balagurunathan
Kubicova, Lenka
Kubik, Stefan
Kubo, Yuji
Kubota, Yasuhiro
Kuca, Kamil
Kucerova, Marta
Kucharikova, Sona
Kucheryavskiy, Sergey
Kuentz, Martin
Kujawski, Jacek
Kukovec, Boris-Marko
Kulakowski, Daniel
Kulkarni, Chandrashekhar V.
Kumar, Gaurav
Kumar, Naresh
Kumar, Sandeep
Kumar, Sumit
Kumbar, Sangamesh G.
Kunz, Wolfram
Kunze, Gotthard
Künzler, Markus
Kuo, Dong-Hau
Kuo, Ping-chung
Küpcü, Seta
Kuramochi, Kouji
Kurdowska, Anna K.
Kurihara, Hideyuki
Kuroda, Minpei
Kurra, Yadagiri
Kurtán, Tibor
Kurteva, Vanya
Kusari, Souvik
Kushida, Hirotaka
Kutner, Andrzej
Kutner, Wlodzimierz
Kuwahara, Masayasu
Kveberg, Lise
Kvítek, Libor
Kwak, Jong-Young
Kwak, Young-Shin
Kwan, Hiu-yee
Kwan, Patrick
Kwasniewski, Misha T.
Kweon, Dae-Hyuk
Kwok, Jessica
Kwon, Oran
Kwon, Sung Won
Kyasa, Shiva Kumar
Kyttaris, Vasileios C.
La Clair, James
La Ferla, Barbara
La, Hyun-Joon
Laali, Kenneth K.
Labate, Joanne A.
Labeda, David P.
Lacaille-Dubois, Marie-Aleth
Lacorn, Markus
Lacrampe, Marie France
Laczkowski, Z.
Lagouri, V.
Laitinen, Riikka
Lallès, Jean-Paul
Lam, Henry
Lam, Kim-Hung
Lamari, Fotini N
Lamberth, Clemens
Lamy, Evelyn
Landais, Yannick
Landete , José Maria
Lang, Christine
Lang, Jian-Ping
Lang, Jochen
Lang, Kai
Lange, Heiko
Langford, Cooper H.
Lante, Anna
Lara-Sanchez, Agustin
Larionov, Oleg V.
Latosińska, Jolanta Natalia
Lattanzi, Alessandra
Lattmann, Eric
Lau-Cam, Cesar A.
Lauer, Mark E.
Lavacchi, Alessandro
Lavandera, Ivan
Lawrence, Glen D.
Lazaridis, Themis
Lazarova, Zelmira
Lazzarato, Loretta
Lazzaroni, Roberto
Le Grognec, Erwan
Leadbeater, Nicholas
Leão, Pedro Nuno da Costa
Lebel, Olivier
Leblanc, Roger
Lebleu, B.
Lecomte, Philippe
Lectka, Thomas
Lederkremer, Gerardo Z.
Lee, Adam
Lee, Adam F.
Lee, Alice
Lee, Bong Ho
Lee, Chang-Hee
Lee, Che-Hsin
Lee, Ching-Kuo
Lee, Choong Hwan
Lee, Dong-seok
Lee, Dong-Ung
Lee, Dong-yup
Lee, Laura
Lee, Eun-Hee
Lee, Heesoon
Lee, Hon Man
Lee, Hye Suk
Lee, Jaehwi
Lee, Jaesung
Lee, Jandee
Lee, Jeong-Ik
Lee, Jeongmi
Lee, Jongkook
Lee, Jooyoung
Lee, Jun
Lee, Jun-Soo
Lee, Kwang Ho
Lee, Kyung-yeol
Lee, Meng-shiou
Lee, Mi Kyeong
Lee, Mijoon
Lee, Min Won
Lee, Moonyong
Lee, Myung Koo
Lee, Ok-Hwan
Lee, Ren-Shen
Lee, Sang Kook
Lee, Sang-han
Lee, Sang-Myung
Lee, Sang-Soo
Lee, Seokjoon
Lee, Seong-Ryong
Lee, Sun-Young
Lee, Taek-Kyun
Lee, Tse-Min
Lee, Tzong-Huei
Lee, Tzong-shyuan
Lee, Won Sup
Lee, Yeon-Ju
Lee, Yong Rok
Lee, Yoon Kwang
Lee, Young Ho
Lei, Aiwen
Leite, Edda Lisboa
Leitner, Erich
Lemus, Jesús
LEN, Christophe
Leo, Chen
León, Rafael
Leonidas, Demetres D.
Leow, Dasheng
Leslie, J.C.
Lesmes, Uri
Lessard, Benoît H.
Lesyk, Roman
Leung, Chung-Hang
Leung, Man-kit
Leung, Yuk-man
Levitskaia, Tatiana G.
Lewinska, Anna
Lewis, Frank W.
Lewis, Lynley K.
Leyva-Pérez, Antonio
Lezot, Frédéric
Li, Bin
Li, Chi
Li, Chunshun
Li, Degang
Li, Dehai
Li, Fengzhi
Li, Hua-Bin
Li, Jie
Li, Jie Jack
Li, Lan-Juan
Li, Li
Li, Menglong
Li, Nianguang
Li, Qian
Li, QingBiao
Li, Qingliang
Li, Quanshun
Li, Shao-Shun
Li, Sheng
Li, Shujun
Li, Shu-Ming
Li, Suming
Li, Wei
Li, Wenhua
Li, Wen-Tai
Li, Xia
Li, Xian
Li, Xiaobo
Li, Xiaohua
Li, Yimeng
Li, Ying
Li, Yonghui
Li, Yuliang
Li, Zhigang
Li, Zhi-Minhg
Liang, Fu-Sen
Liang, Pi-Hui
Liang, Yong
Lianos, Panagiotis
Liao, Yong-Hong
Liaw, Chih-Chuang
Liburdi, K.
Lichtenstein, Rachel G.
Lidon, Fernando Cebola
Liebscher, Juergen
Liégeois, Jean-François
Liekens, Sandra
Liermann, Johannes C.
Lill, Markus A.
Lim, Chee
Lim, Sang-bin
Lim, Soon Sung
Lim, Yoongho
Lima, Cristovao F.
Lima-Brito, José Eduardo
Lima-Neto, Benedito
Limbeck, Andreas
Limnios, Dimitris
Lin, Chih-Chien
Lin, Chih-Yuan
Lin, Chun-Jung
Lin, Hong-Liang
Lin, Hung-Yin
Lin, Jianguo
Lin, Jing-jer
Lin, Jong-Liang
Lin, JunFang
Lin, Lianzhu
Lin, Li-Huei
Lin, Mei-hsiang
Lin, Shuhai
Lin, Yu-Hsin
Lin, Yun-wei
Lindstrom, Jon Martin
Linert, Wolfgang
Ling, Jun
Ling, Tie-Jun
Linhardt, Robert
Link, Wolfgang
Lionetti, Vincenzo
Lipinski, Boguslaw
Lisanti, M.T.
Lisewski, Andreas Martin
Lissalde, Sophie
Litaudon, Marc
Litinas, Konstantinos E.
Little, R. Dan
Litvić, Mladen
Liu, Baoming
Liu, Bifeng
Liu, Cheuk Lun
Liu, Chong
Liu, Chong-Huai
Liu, Donghong
Liu, Fang
Liu, Hai-yang
Liu, Hui-Hsuan
Liu, Jian
Liu, Jiemin
Liu, Junwen
Liu, Liang
Liu, Qingsong
Liu, Shing-Hwa
Liu, Shiuh-Tzung
Liu, Shiyong
Liu, Shulin
Liu, Wangqing
Liu, Xiaojing
Liu, Xiaoming
Liu, Xiaoxi
Liu, Xinghai
Liu, Xinyong
Liu, Yang
Liu, Yingqian
Liu, Yonghong
Liu, Yongqiang
Liu, Zhijun
Liu, Zhi-Long
Liu, Zhongqiu
Livera, Alysha M. De
Llebaria, A.
Llona-Minguez, Sabin
Llop, Jordi
Lo Piero, Angela Roberta
Lo Scalzo, Roberto
Lo, Clive
Lobo Castañón, Maria Jesus
Locatelli, Erica
Locatelli, Marcello
Lohberger, Birgit
Lok, Chun Nam
Lomascolo, Anne
Lombó, Felipe
Long, Chunlin
Longo, Vincenzo
Lönnberg, Harri
Loo, Rachel
López, Carmen Blanco
López, María L.
Lopez, Moisés Canle
Lopez, Paloma
Lopez-Jaramillo, Francisco Javier
López-Miranda, Visitación
Lórenz-Fonfría, Víctor A.
Lorenzi, V.
Lortat-Jacob, Hugues
Lou, Bih-Show
Lou, Ching-Wen
Lou, Y.J.
Lu, Gang
Lu, Guifen
Lu, Lvqun
Lu, Wei Yue
Lu, Xing
Lu, Yan-Hua
Lu, Yi
Lucas, Guilherme
Lucena, R.
Lüchow, Arne
Lüddens, Hartmut
Ludidi, Ndiko
Ludwiczuk, Agnieszka
Lugo-Cervantes, Eugenia
Lui, Edmund M.K.
Luis, Santiago V.
Luisi, Renzo
Lukyanov, Pavel A.
Luna, Amparo
Luna, Jonny E. Duque
Lung, Ming-Yeou
Luo, Minkui
Luo, Yinggang
Luo, Zisheng
Luong, Dzung Dinh
Luongo, Livio
Lupiáñez, José A.
Lurie, Susan
Luxen, André
Lvov, Yuri
M. Graça Dias, M. Graça
Ma, Chao-Mei
Ma, Dik-Lung
Ma, Jin Yeul
Ma, Lin
Ma, Shengqian
Ma, Xiaoqian
Ma, Yongjie
Ma, Zhongjun
Maccallini, Cristina
Macchi, Beatrice
Machado, Vanderlei G.
Machetti, Fabrizio
Machut-Binkowski, Cécile
Macias Sanchez, Antonio José
Maciejewska, Dorota
Mackerell, Alexander D
Mackowski, Sebastian
Maçôas, Ermelinda
Macovei, Anca
Macquarrie, Duncan
Mączka, Mirosław
Madder, Annemieke
Madesis, Panagiotis
Madiraju, S.R. Murthy
Mäeorg, Uno
Maestro, Miguel
Magata, Yasuhiro
Maggi, Filippo
Maggio, Antonella
Magiatis, Prokopios
Magid, Abdulmagid Alabdul
Mahalingam, Marthandan
Maia, José Guilherme S.
Maier, Robert J.
Maione, Francesco
Maisch, Tim
Majoumerd, Mohammad Mansouri
MAK, King Lun Kingston
Makabe, Hidehumi
Mäkelä, Miia R.
Makishima, Makoto
Makris, Dimitris P.
Malaguarnera, Michele
Malaguti, Marco
Malheiro, Ricardo
Malitesta, Cosimino
Malkoch, Michael
Malkowski, Michael G.
Mallem, Mohamed Yassine
Malli, Roland
Mallipeddi, Prema Latha
Malloci, Giuliano
Mambu, Angele
Mandal, Debashis
Mandrone, Manuela
Mañes, Jordi
Mannelli, Lorenzo Di Cesare
Mannino, Saverio
Manns, David Christopher
Manso, S.
Manthey, John A.
Manthina, Venkata
Mantle, Michael D.
Manz, Thomas A.
Manzhos, Sergei
Mao, Bin
Mao, Zong-Wan
Mar Contreras, María Del
Maracaja-Coutinho, Vinicius
Maraldi, Tullia
Marchand, Pascal
Marco-Contelles, José
Marcotullio, Maria Carla
Maréchal, Jean-Didier
Margetić, Davor
Maric, Milan
Marik, Jan
Mario, Dellagli
Markiewicz, Wojciech T.
Markopoulos, John
Markopoulou, Olga
Markuszewski, Michal J.
Maroteaux, Luc
Marriott, Karla
Marrucho, Isabel
Marsaioli, Anita Jocelyne
Marsik, Petr
Martel, Fátima
Martelli, Pier Luigi
Martic, Sanela
Martí-Gastaldo, Carlos
Martín, M. Elena
Martin, Rainer
Martinelli, Adriano
Martínez, Ana
Martínez, Eva Garcia
Martinez, Gary V.
Martínez-Mayorga, Karina
Martínez-Palou, Rafael
Martínez-Soria, V
Martinho, J.M.G.
Martin-Ortigosa, Susana
Martins, João B.L.
Martins, Luísa M.D.R.S.
Martins, M. Cristina L.
Martins, Maria do Rosário
Martins, Viviana
Martin-Sampedro, Raquel
Martinussen, Inger
Maruyama, Kei
Marz, Manja
Masaki, Hitoshi
Masaoka, Shigeyuki
Mase, Nobuyuki
Masetti, Matteo
Massa, Antonio
Masters, Colin L.
Mas-Torrent, Marta
Masuda, Shinji
Masutani, Hiroshi
Matés, José M.
Mathema, Vivek Bhakta
Mathesius, Ulrike
Mathew, Thomas
Mathieu, Didier
Matsoukas, John
Matsuda, Hisashi
Matsuda, Yudai
Matsui, Masaki
Matsui-Yuasa, Isao
Matsumura, Kiyoshi
Matsumura, Shuichi
Matsuo, Takashi
Matsuya, Yuji
Matsuzaki, Yasushi
Mattevi, Andrea
Maurer, Andreas
Maves, Lisa
Mavri-Damelin, Demetra
Mavromoustakos, Thomas
Mayence, Annie
Mazzeo, Marco
Mazzone, Gloria
Mazzoni, I.
Mazzoni, Luca
McAlpine, James B.
Mcardle, Patrick
McCallum, Jason L.
McCarty, Douglas M
Mcconnell, Michelle
McConville, Christopher
Mcconville, Malcolm
McCormick, Alon V.
Mcdougall, Gordon J.
Mcdowell, Arlene
McFeeters, Robert L.
McGarrigle, Eoghan M.
Mcginley, John
Mcmahon, Robert J.
Mechler, Adam
Medeiros Ferreira, João Paulo
Medina, Miguel Angel
Medronho, Bruno
Mehlhorn, Heinz
Mehta, Akul Y.
Mei, Nan
Mei, Wenli
Mei, Xuefeng
Meierjohann, Svenja
Meijden, Maarten Van Der
Melchers, Robert
Mele, Giuseppe
Melucci, Dora
Melzig, Matthias
Mena, Pedro
Mena-Rejon, Gonzalo J.
Menezes, José
Meng, Fenghua
Meng, Menghsiao
Meng, Qingxiong
Menna, Marialuisa
Menzel, Gherard
Merah, Othmane
Mercer, John R.
Mercolini, Laura
Mergny, Jean-Louis
Merino, Pedro
Merlini, Lucio
Merlino, Antonello
Mertz, Christian
Messori, Luigi
Mestres, Adrià Gil
Metzinger, Laurent
Meyer, Hans-Peter
Meyer, Torsten
Meyerhof, Wolfgang
Meyers, Marvin J.
Miao, Jun-ying
Micale, Nicola
Miccadei, Stefania
Michaelakis, Antonios
Michalski, Stefan Georg
Michel, Sarah L.J.
Micucci, Matteo
Micura, Ronald
Middelkoop, Vesna
Miele, Adriana E.
Mier, Walter
Mignani, Anna Grazia
Mignet, Nathalie
Miguel, Daniel
Miguel, Graça
Mikstacka, Renata
Mikulski, Damian
Milelli, Andrea
Miller, Alain O.A.
Miller, Daniel G.
Millo, Enrico
Mimaki, Yoshihiro
Mimica-Dukic, Neda
Min, Sun-Joon
Minami, Ysunori
Minassi, Alberto
Minbiole, Kevin P.C.
Minden, Jonathan S.
Minero, Claudio
Miranda, Katrina
Mirjafari, Arsalan
Mishra, Yogendra K.
Misochko, E. Ya.
Misra, Biswapriya B.
Mitakou, Sofia
Mitchell, Julie C.
Mitra, Abhisek
Mitraki, Anna
Miyabe, Hideto
Miyamoto, Yohei
Miyata, Yoshihiko
Miyazaki, Daigo
Mizuno, Cassia S
Mladěnka, Přemysl
Mladenović, Milan
Mlcek, Jiri
Młochowski, Jacek
Moberg, Christina
Mody, Karsten
Mohammad, Altaf
Mohan, Tamilselvan
Mohanam, Sanjeeva
Mohanta, Tapan
Mohr, Fabian
Moineau-Chane-Ching, Kathleen I.
Mok, Daniel Kam-Wah
Molinari, Alessandra
Molinari, Maurizio
Mollapour, Mehdi
Molnár, Ferdinand
Molnar, Maja
Monflier, Eric
Monguchi, Yasunari
Monobe, Hirosato
Monopoli, Antonio
Monrad, Rune Nygaard
Montenegro, Lucia
Montoro, Paola
Moo-Huchin, Víctor M.
Moon, Dohyun
Moon, Hyung Ryong
Moon, Hyung-In
Moon, Kyoung-Sik
Moon, Seung-hyeon
Moon, Yong-Hwan
Moorthy, N.S. Hari Narayana
Morales, Jose
Moran, Grainne
Morán, Lara
Morata, Antonio
Morato, Manuela
Moravcová, Dana
Morawski, Antoni W.
Moreno, J.J.
Moreno, Silvia
Morgan, Alexander
Morgan, Donnard
Morikawa, Hisao
Morisseau, Christophe
Morita, Kyoji
Moriyama, Katsuhiko
Morre, James
Morreale, Giacomo
Morsi, Badie
Mortier, Jérémie
Morvan, Francois
Morzherin, Yury
Mosset, Paul
Motilva, Maria-José
Motta, Andrea
Motta, Concettina La
Mouchlis, Varnavas D.
Mouloungui, Zéphirin
Mouret, Jean-Roch
Mourtzinos, Ioannis
Moutinho-Pereira, José Manuel
Moyano, Albert
Moyano, Lourdes
Mozzon, Massimo
Mphahlele, Malose Jack
Muccioli, Giulio G.
Mukhar, Hasan
Mukudai, Yoshiki
Muller, C.J.F.
Müller, Christian P.
Müller, Karsten
Müller, Sylke
Müller, Thomas J.J.
Mulloy, Barbara
Mun, Jiyoung
Munang’andu, Hetron M.
Muppidi, Avinash
Murafuji, Toshihiro
Murai, Masahito
Murai, Toshiyuki
Murakami, Kazuma
Murata, Kenji
Murata, Toshihiro
Murata, Yoshinori
Murato, Miguel
Murelli, Ryan P.
Murkovic, Michael
Muro, Silvia
Murota, Kaeko
Murray, Deirdre M.
Murray, Jane S.
Musci, M.
Musiol, Robert
Musso, Loana
Mustafa, Arwa
Mutelet, Fabrice
Muthaiyan, Arun
Mutus, Bulent
Myers, Linda K.
Nagai, Kouhei
Nagai, Ryoji
Nagai, Yoshinori
Nagamitsu, Tohru
Nagaoka, Satoshi
Nagasawa, Kazuo
Nagata, Eiichiro
Nagatani, Hirohisa
Nagatomo, Shigenori
Nagendra Reddy, Chintam
Nagl, Marcus
Nair, Vimal
Nakagawa, Yoshiaki
Nakamura, Akinori
Nakamura, Itaru
Nakamura, Kazufumi
Nakamura, Kozo
Nakanishi, Ikuo
Nakase, Ikuhiko
Nakata, Satoshi
Nam, Ki Tae
Nam, Sang-Jip
Nanda, Kaushik
Nantasenamat, Chanin
Naoi, Makoto
Napoleão, Thiago Henrique
Napoletano, Chiara
Nascimento, Alessandro S.
Natarajan, Amarnath
Navarrete, Gabriel
Navrot, Nicolas
Nazaruk, Jolanta
Nazzal, Sami
Nazzaro, Filomena
Neely, Brian J.
Negri, Giuseppina
Nehdi, Imededdine Arbi
Nehira, Tatsuo
Nemykin, Victor
Nemzer, Boris
Nenadis, Nikolaos
Nesnas, Nasri
Nesterova, Irina V.
Neugart, Susanne
Neumann, Donna M.
Nevárez-Moorillón, Guadalupe Virginia
Neves, M. Graça P.M.S
Newman, David
Ng, Danny Wang Kit
Ng, Ken
NG, Kevin Tak-Pan
Nguyen, Phuong
Nguyen, Thanh Binh
Ni, Bukuo
Nicasio, M. Carmen
Nicklaus, Marc C.
Nicolaou, K.C.
Nielsen, Kristian Fog
Nielsen, Line Hagner
Nieminen, Kaarlo
Niessen, Ludwig
Nihei, Ken-ichi
Nikolic, Dejan
Ninfali, Paolino
Nishibayashi, Yoshiaki
Nishida, Yoshihiro
Nishihara, Yasushi
Nishikiori, Hiromasa
Nishiwaki, Nagatoshi
Niu, Xiao-Feng
Nobili, Stefania
Noda, Naohiro
Noè, Marco
Noge, Koji
Nogueira, Cristina
Noiseux, Nicholas
Nokami, Toshiki
Nomikos, Tzortzis
Nomiya, Takuma
Noonan, Kevin
Nordstierna, Lars
Norton, Michael
Norval, Graeme W.
Norvez, Sophie
Nosova, Emiliya V.
Novak, Johannes
Novak, Lajos
Nowak, Sascha
Ntalli, Nikoletta G.
Nunes, Fábio Souza
Nyulász, László
O. Sintim, Herman
O’brien, Peter
O’Maille, Paul E.
O’Shea , Kevin E.
Oancea, Elena
Oberholster, Anita
Obied, Hassan
O'Connor, Sarah
Odell, Luke R.
O'Doherty, George
Ogata, Tadanori
Ogawa, Akiya
Ogihara, Takuo
Ogungbe, Ifedayo Victor
Oh, Dong-Chan
Oh, Jae-Min
Oh, Tae-Jin
Ohkohchi, Nobuhiro
Ohlendieck, Kay
Ohnishi, Masatoshi
Ohno, Sho
Ohta, Masaya
Ohta, Shigeo
Ohtake, Toshihiro
Oikawa, Shinichi
Ojika, Makoto
Ok, Salim
Okada, Taketo
Okafor, Florence
Okamura, Katsutomo
O'Keefe, Barry R.
Okino, Tatsufumi
Okuma, Kentaro
Olas, Beata
Oliveira, Rui
Oliveras-López, María-Jesús
Oliverio, Manuela
Ollis, David
Ong, Eng Shi
Onnis, Valentina
Ono, Masateru
Oostenbrink, Chris
Oppelt, Kerstin T.
Oppenheimer, Steven B.
Orduna, Jesús
O'Reilly, Elaine
Oreopoulou, Vassiliki
Orian, Laura
Ortega, Angel L.
Ortelli, Simona
Ortiz, Alfredo
Osawa, Tsutomu
Osborn, Helen
Oshitari, Tetsuta
Osorio Rosa, Coralia
Osorio, Edison
Østergaard, Jesper
Ostrowski, Stanisław
Ota, Akinobu
Otagaki, Shungo
Otagiri, Masaki
Otsuka, Hideaki
Otsuka, Masami
Ottmann, Christian
Ottolina, Gianluca
Oufir, Mouhssin
Ouyang, Ming-An
Ouzounidou, Georgia
Owen, Shawn C.
Owttrim, George
Oyama, Masayoshi
Oyoshi, Takanori
Ozaki, Hiroaki
Ozoe, Yoshihisa
Pacetti, Debora
Paci, M.
Pacifico, Severina
Padilla-Martínez, Itzia I.
Padrela, Luis
Pae, Ae Nim
Paiva-Martins, Fátima
Pajeva, Ilza
Pal, Rituraj
Palacios, Sara
Palazzo, Enza
Palenzuela López, José Antonio
Paliy, Oleg
Palmer, Melody R.
Palomba, Letizia
Palomo, G. Iván
Palou, Lluís
Palza, Humberto
Pan, Caiyuan
Pan, Po-Shen
Pan, Rong-long
Pan, Tai-long
Panaccione, Daniel G.
Panayiotou, Costas
Panfoli, Isabella
Panuwet, Parinya
Panzella, Lucia
Paoli, Paola
Papachristos, Dimitrios P.
Papadoyiannis, Ioannis N.
Papetti, Adele
Papini, Emanuele
Papy-Garcia, Dulce
Parac-Vogt, Tatjana N.
Parente, Augusto
Park, Hae Ryoun
Park, Il-Kwon
Park, In-Kyu
Park, Jin Kyoon
Park, Ji-yeon
Park, Jong-Chul
Park, Sehoon
Park, Seung-chun
Park, Tae Joo
Park, Wansu
Parkinson, Don-Roger
Parrino, Francesco
Paschke, Angelika
Pascu, Mihail Lucian
Pascual, Alberto
Pascual-Teresa, Sonia
Pasqua, G.
Passarella, Daniele
Passchier, Jan
Pastor, F.J.
Pastore, Mariachiara
Patel, Hardikkumar
Patel, Mrudula
Pathuri, Gopal
Pati, Sandra
Paton, Robert S.
Patsenker, Leonid
Patzak, Josef
Paudel, Liladhar
Paula, Stefan
Paull, Jeffrey
Paulsen, Berit
Pavan, Fernando R.
Pavela, Roman
Pawlaczyk, Izabela
Pearson, Jason
Pecinka, Ales
Peddabuddi, Gopal
Pedneault, Karine
Pedraza-Chaverri, José
Pedretti, Alessandro
Pedrosa, Rafael
Peinado, Juan R.
Pék, Zoltán
Pellerito, C.
Pelletier, Guillaume
Peña, Diego
Pena-Pereira, Francisco
Pendleton, Phillip
Peng, Chiung–Huei
Peng, Robert Y.
Peng, Ruogu
Peng, Wen-Huang
Penmetcha, Kumar
Pereira, C.
Pereira, Cynthia L.M.
Pereira, Florbela
Pereira, Leonel
Pereira, Olívia R.
Pereira, Vanda
Perera, Ana
Peretz, Avi
Pérez, Andy J.
Perez, Lark
Perez, Pedro J.
Perez-Benito, Joaquin F.
Perissutti, Beatrice
Perpète, Eric A.
Perticone, Francesco
Petersen, Maike
Petersen, Mikael Agerlin
Peterson, Steven C.
Petitjean, Anne
Petrat-Melin, Bjørn
Petretto, Giacomo L.
Petrozziello, Maurizio
Pezzella, Alessandro
Pfaller, M.A.
Pferschy-Wenzig, Eva-Maria
Pham, Ngoc
Philippe, Cécile
Philips, Neena
Pi, Rongbiao
Piaz, Fabrizio Dal
Pichat, Pierre
Picot, L.
Piel, Markus
Pierens, Gregory K.
Pihlanto, Anne
Pii, Youri
Pike, Victor W.
Pilc, Andrzej
Pillay, Viness
Pilu, Roberto
Piñeiro Gomez, Marta
Pinheiro, Carla
Pino, Verónica
Pinteala, M.
Pinto, Diana C.G.A.
Piosik, Jacek
Piotrowski, Piotr
Pires, E.
Pires, José Carlos Magalhães
Pitsikalis, Marinos
Pitucha, Monika
Pizzimenti, Stefania
Planas, Marta
Plano, Daniel
Pla-Quintana, Anna
Plastina, Pierluigi
Plech, Tomasz
Pleissner, Daniel
Poater, Albert
Poce, Giovanna
Poczai, Péter
Podany, Anthony T.
Poddel'sky, Andrey I.
Pogoń, Krystyna
Poinsot, Verena
Poleszak, Ewa
Politeo, O.
Polticelli, Fabio
Polyak, Stephen J
Polyak, Steven
Polyakov, N.E.
Poni, Stefano
Pons, Josefina
Pontiki, Eleni
Popat, Amirali
Popiołek, Łukasz
Popov, Alexey A.
Popov, Anatoliy V.
Porcheddu, Andrea
Poroikov, Vladimir V.
Portaccio, Marianna
Pospieszny, Tomasz
Potterat, Olivier
Poulas, Konstantinos
Poulos, Thomas L.
Poulsen, Anders
Poulsen, Thomas B.
Pourquier, Philippe
Poyatos, Macrena
Pradeepkumar, P.I.
Prado, Soizic
Praena, Daniel Gutierrez
Prandi, Cristina
prasanth Koppolu, Bhanu
Prat, Maria
Preston, Christopher
Preti, Raffaella
Priefer, Ronny
Priemel, Petra
Prieto, Jose M.
Prieto, M. Auxiliadora
Prieto-Garcia, Jose M.
Prinsloo, Earl
Procopio, Susanne
Proestos, Charalampos
Prohens, Rafel
Pronin, Alexander V.
Psarra, Anna-maria G.
Pu, Lin
Pu, Xiaoping
Puddephatt, Richard
Puerta, M. Carmen
Pulati, Nuerxida
Püschel, Gerhard P.
Putz, Eva Maria
Pyka, Alina
Qi, Phoebe X.
Qian, Bing-Jun
Qian, Mingxing
Qian, Ziqing
Qiao, Chunhua
Qin, Xuemei
Qiu, Ming-hua
Quan, Zhe-Shan
Quax, Wim J.
Queiroz, Maria-João R.P.
Quinonero, David
Quintard, Jean-Paul
Quirino, Joselito P.
Raabe, Gerhard
Rademacher, Christoph
Radziuk, Darya V.
Raes, Katleen
Rafii, Fatemeh
Ragauskas, Art J.
Ragno, Rino
Raguso, Robert
Rai, Dilip K.
Raja, Krishnaswami
Rakotondraibe, L.H.
Raliya, Ramesh
Ralston, Katherine
Ramidi, Punnamchandar
Ramirez, Anibal Maury
Ramos, Freddy A.
Ramos, Sonia
Ramos-Parra, Perla
Ramozzi, Romain
Rampino, Sergio
Ramsay, Rona
Ramtoola, Zebunnissa
Raner, Gregory M.
Ranzato, Elia
Rao, Juluri R.
Rao, P.N. Praveen
Rao, Yu
Raposo, Manuela
Rapp, Michael
Rasulev, Bakhtiyor
Ratnadass, Alain
Ratz-Łyko, Anna
Raucher, Drazen
Rawel, Harshadrai
Ray, Adrian S.
Ray, Partha
Raynes, J.K.
Reany, Ofer
Rebane, Aleksander
Rebl, Alexander
Rebolledo, Francisca
Rebollo, Angelita
Reddy, Narsa M.
Reebye, Vikash
Reese, R. Neil
Reese, Torsten
Reetz, Manfred T.
Reeve, Amy
Reeve, Vivienne
Regel-Rosocka, Magdalena
Réglier, Marius
Reichardt, Niels-Christian
Reinders, Joerg
Reiser, Oliver
Reisman, Scott A.
Reitzel, Lotte A.
Reliene, Ramune
Remko, Milan
Ren, Hongjun
Ren, Jian-guo
Ren, Junli
Ren, Lei
Ren, Shuxin
Ren, Xuehong
Renaut, SÉbastien
Renner, Ulrich
Restani, Patrizia
Revilla, Eugenio
Reyes, Fernando
Reynisson, Jóhannes
Ricardo, Reyes-chilpa
Ricca, Ezio
Ricci, Alfredo
Ricciardi, Giampaolo
Ricciotti, L.
Riela, Serena
Rietveld, Ivo B.
Riguera, Ricardo
Rimondini, Lia
Riou, Laurent M.
Ritchie, David W.
Rivara, Silvia
Rivas, Fatima
Rizos, Helen
Ro, Dae-Kyun
Roberto, Dominique
Robertson, Mark J.
Robins, Joe
Robinson, Ross
Roccatano, Danilo
Rocha, Joao B.T.
Rocha-Guzmán, Nuria Elizabeth
Roche, Daniel
Rocheleau, Jonathan V.
Rodilla, Jesus M.
Rodrigues, António S.
Rodrigues, Rodney A.F.
Rodríguez, Raúl
Rodríguez-Gonzalo, Encarnación
Rodríguez-López, Julián
Roebuck, Bill
Roesch, Frank
Rojo, Javier
Roland, Aurélie
Rolle, Luca
Romero, Alberto
Rosa, Joaquín
Roseiro, Luísa B.
Rosete, Maria Dra Teresa Cruz
Ross, Matthew
Ross, Samir
Rotenberg, Susan A.
Rotureau, Patricia
Rouille, Yves
Routray, Winny
Roy, Denis
Roy, Rene
Różalska, Barbara
Rozanska, Xavier
Rozas, Isabel
Rozec, Bertrand
Ruban, Alexander
Rubió, Laura
Rubio, Soledad
Rubis, Blazej
Rucker, Robert B.
Rudick, Jonathan
Rudnicki, Dobrila Doda
Ruediger, Hell
Ruffoni, Barbara
Ruggerone, Paolo
Ruijter, Eelco
Ruiz-Aceituno, Laura
Ruiz-Romero, Cristina
Rupasinghe, Vasantha
Rurali, Riccardo
Rus, Alma
Russo, Maria
Russo, Nino
Russowski, Dennis
Russu, Wade A.
Rutjes, Floris P.J.T.
Ryu, Chung-kyu
Ryu, Jae-Ha
Ryu, Jong Hoon
Ryu, Young Bae
S. Silva, M. Luísa
Sabila, Paul Sali
Sabo-Attwood, Tara
Sabot, Cyrille
Sacks, Gavin
Sackus, Algirdas
Sadekar, Shraddha
Sadowska, Beata
Sahlstrøm, Stefan
Said, Ahmed M.
Saito, Akio
Saito, Hiroshi
Sakaguchi, Shota
Sakai, Satoshi
Sakakibara, Hiroyuki
Sakakura, Akira
Sakurai, Takuya
Salaün, Fabien
Saleh, Mahmoud A.
Salgado, Antonio
Salgueiro, Ligia
Salinas, Rosario
Saluk-Juszczak, Joanna
Salvatore, Ralph Nicholas
Salvia-Trujillo, Laura
Salvio, Riccardo
Samovski, Dmitri
Samuel, Temesgen
San Martin, Carmen
Sanchez, Goar
Sanchez-Cortes, Santiago
Sánchez-Moreno, Manuel
Sánchez-Vioque, R.
Sancini, Giulio
Sandell, Johan
Sandwick, Roger
Sang, Shengming
Sangadala, Sreedhara
Sangaraju, Dewakar
Sanjust, Enrico
Sanny, Charles G.
Sansano, José M.
Sant, Marco
Santagati, Andrea
Santi, Claudio
Santillo, Michael
Santos, Clementina
Santos, Sónia A.O.
Santulli, Gaetano
Sarafidis, Kosmas
Saris, Wim
Sarkar, Biprajit
Sarker, Satyajit D.
Sarrazin, Sandra Layse Ferreira
Sasson, Yoel
Sastre-Santos, Angela
Šatínský, Dalibor
Sato, Hirotaka
Sato, Kenji
Sato, Yuichiro
Satoh, H.
Satomi, Yoshiko
Saturnino, Carmela
Saulnier, Luc
Savaskan, Nic E
Savio, L.
Savoia, Dianella
Savoia, Diego
Savoie, Jean-Michel
Saxena, Praveen
Sayago, Ana
Sberveglieri, Veronica
Scarlett, Christopher
Scendo, Mieczyslaw
Schaberle, Till F.
Schäfer, Michael
Schalkwijk, Casper G.
Schauss, Alexander G.
Scheidt, Holger A.
Scheiner, Steve
Schepdael, Ann Van
Scherm, Harald
Schiffman, Jessica D.
Schinkovitz, Andreas
Schlyer, David
Schmeda-Hirschmann, Guillermo
Schmid, Markus
Schmidt, Andreas
Schmidt, Boris
Schmidt, Jürgen
Schmidt, Martin U.
Schmidt, Olaf
Schnackenberg, Laura
Schneekloth, Jr., John S.
Schneider, Bernd
Schneider, Christoph
Schneider, Claus
Schnermann, Martin J.
Schubert, Mario
Schueffler, Anja
Schuffenhauer, Ansgar
Schuhmacher, Rainer
Schulz, Benjamin L.
Schulz, Hartwig
Schulzová, Věra
Schurig-Briccio, Lici
Schurigt, Uta
Schuster, Bernhard
Schwartz, Nancy B.
Ścianowski, Jacek
Scognamiglio, Monica
Scott, Jason
Sébédio, Jean-Louis
Šebesta, Radovan
Seca, Ana M.L.
Seeger, Christoph
Seela, Frank
Segal, Mark S.
Seger, Christoph
Sehgal, Alfica
Seidel, Véronique
Seidl-Seiboth, Verena
Seifert, Karlheinz
Seio, Kohji
Seley Radtke, Kathie
Sellam, Adnane
Sémeril, David
Sendker, Jandirk
Seo, Woo Duck
Seppälä, J.V.
Seppälä, Jukka
Serna-Saldívar, Sergio O.
Serra, Immacolata
Serra, Stefano
Serrano, María
Sestili, Piero
Setzer, William
Seubert, John M.
Seviour, Robert J.
Seybold, Paul G.
Sgarbossa, Paolo
Shah, Gul N.
Shah, Zahoor
Shahbazi, Mohammad Ali
Shainyan, B.A.
Shan, Anshan
Shang, Dejing
Shang, Ming-Ying
Shankar, Eswar
Shao, Changlu
Shao, Fangwei
Shao, Lei
Sharma, Arun K
Sharma, Sherven
Sharon, Ashoke
Shavrukov, Yuri
Shaw, Anthony
Shaw, Jrard
Sheibani, Nader
Sheldon, Julie
Sheldrake, Gary
Shen, Jian Gang
Shen, Jingshan
Shen, Qi
Shen, Shihua
Shen, Xiaoqin
Shen, Ya-Ching
Shen, Yingjia
Shen, Yongjia
Shen, Yue-Mao
Sheng, Rong
Sheng, Yin
Sheu, Joen R.
Sheu, Jyh-Horng
Shi, Dayong
Shi, Feng
Shi, Hua
Shi, Jie-hua
Shi, Yawei
Shi, Zhan
Shia, Kak-shan
Shiau, Chung-Wai
Shibasaki, Yuji
Shibata, Ikuya
Shibata, Norio
Shibuya, Masatoshi
Shiea, Jentaie
Shieh, Tzong-Ming
Shigdar, Sarah
Shigemori, Hideyuki
Shih, Ing-Lung
Shih, Mei-Hsiu
Shih, Tzenge-Lien
Shih, Wen-Ling
Shimamura, Yuko
Shimizu, Karina
Shimizu, Yo
Shimizu, Yohei
Shimoda, Hiroshi
Shin, Chanseok
Shin, Damian S.
Shin, Dong-Soo
Shin, Sung Chul
Shinn, sara
Shiraishi, Yasuhiro
Shirakami, Yohei
Shirasawa, Kenta
Shirure, Venktesh
Shityakov, Sergey
Shiue, Yow-ling
Shmizu, Ken
Shu-Huei, Kao
Shukla, Shivendra D.
Shul'pin, Georgiy
Si, Hongwei
Si, Tong
Sicher, Richard C.
Siculella, Luisa
Siedlecki, Paweł
Sikora, Ewa
Sikva, Artur M.S.
Silaghi-Dumitrescu, Radu
Silipo, Alba
Silva , Bagnólia A.
Silva, Ana Rosa
Silva, Fátima Regina Mena Barreto
Silva, Filomena
Silva, João
Silva, Luís R.
Silva, Maria Fátima
Silva, Olga
Silva, Sónia
Silvestre, Armando J.D.
Silvestri, Romano
Simin, Natasa
Simirgiotis, Mario J.
Simmonds, Monique S.J.
Simmons, Katie
Simões, Isaura
Simone, Giuseppina
Simón-Fuentes, Antonio
Simonsen, Henrik Toft
Simpson, David A.C.
Simpson, Garth J.
Simpson, Jim
Since, Marc
Singh, Ashutosh
Singh, Harkesh B.
Singh, Manish
Singh, Nisha
Singh, Udai P.
Sinicropi, Maria Stefania
Siodłak, Dawid
Siracusa, Laura
Sirci, Francesco
Sirimulla, Suman
Siroma, Z.
Sitko, Rafal
Siwek, Agata
Sjogren, Klara
Skalicka-Woźniak, Krystyna
Skaltsa, Helen D.
Skenderi, Katerina P.
Skoda-Földes, Rita
Sladkov, Vladimir
Slama, James T.
Sławiński, Jarosław
Slobbe, Paul
Slootweg, J.C.
Smagghe, Guy
Smani, Younes
Smart, Christine D.
Smeekens, Sanne Petronella
Smeets, Karen
Šmejkal, Karel
Smid, Scott D.
Smit, Andries J.
Smith, Ernest E.
Smith, Len
Smith, Paul A.
Smoleński, Piotr
Smolinska, Agnieszka
Snyder, James P.
Snyder, Scott Alan
Sobarzo-Sánchez, Eduardo
Sobczak, Marcin
Sobkowski, Michal
Sobolev, Anatoly P.
Sobrio, F.
Södergård, Anders
Soengas, Raquel
Soeta, Takahiko
sohn, youngku
Sokolove, Jeremy
Šolaja, Bogdan A.
Soliman, Mahmoud E.S.
Solioz, Marc
Son, Chang-Gue
Sone, Hideko
Song, Baoan
Song, Kenan
Song, Sang Hoon
Song, Yang-heon
Song, Yanlin
Song, Zhihong
Sonnino, Sandro
Soo, Tan Kai
Soong, Ronald
Sørensen, Jens Laurids
Soreq, Hermona
Sorokin, A.B.
Sosa, Silvio
Soto, Carmen
Sousa, Carla
Sousa, Maria C.
Sousa, Sérgio Filipe
Souza, Maria A.
Spagnuolo, Carmela
Spagnuolo, P.A.
Spalletti, Anna
Spanakis, Marios
Spanier, Britta
Špánik, Ivan
Spano, Giuseppe
Sparatore, Anna
Späth, Andreas
Specker, Edgar
Spigoni, Valentina
Spremo-Potparević, Biljana
Sprynskyy, Myroslav
Srivastava, Vibha
Srivatsan, Eri S.
Sroka, Zbigniew
Srzednicki, George
Stacey, Gary
Stadler, Marc
Staege, Martin S.
Stahl, Frank
Stamatatos, Theocharis C.
Staniszewska, Monika
Staples, Richard J.
Stapleton, P.A.
Stasch, Andreas
Stathopoulos, Costas
Stefanachi, Angela
Stefanovic, Darko
Steinberg, Francene M.
Steindel, Mário
Steinfeldt, Norbert
Steven, Sebastian
Stevenson, Bradley
Stewart, Derek
Stewart, Michael W.
Stiger-Pouvreau, Valérie
Stine, Keith J.
Stiriba, Salah-Eddine
Stocker, Bridget
Stojanovic, Ivana
Stoykovich, Mark P.
Strand, Daniel
Strano, Michael S.
Strekowski, Lucjan
Striegel, André M.
Strout, Douglas L.
Stuppner, Hermann
Stura, Enrico A.
Sturini, Michela
Su, Zi-Ren
Subramaniam, Prasad
Sueda, Takuya
Suetake, Isao
Suga, Seiji
Sugahara, Takuya
Sugai, Takeshi
Sugawara, Akira
Sugaya, Nobuyoshi
Sugimoto, Sachiko
Sugiura, Yoshimasa
Suh, Hyung Joo
Suib, Steven L.
Suisse, Isabelle
Suklje, Katja
Sun, Bai-Wang
Sun, Daekyu
Sun, Dequn
Sun, Grace
Sun, Jianwei
SUN, LITAO
Sun, Luyi
Sun, Peilong
Sun, Runcang
Sun, Zhihua
Sun, Zhonghua
Sunatsuki, Yukinari
Sung, Mi Jeong
Sung, Ping-Jyun
Sutherland, Hamish S.
Sutherland, John B.
Sutherland, Todd
Suzuki, Masahito
Suzuki, Tadashi
Suzuki, Tomohiro
Suzuki, Yumiko
Sweazea, Karen
Swiegers, Jan Hendrik
Swift, Jennifer A.
Szczepanik, Maryla
Sze, Marc
Szeto, Yim Tong
Szeto, Yim-Tong
Szöllősi, Dániel
Sztanke, Małgorzata
Szumny, Antoni
Szunerits, Sabine
Tabarrini, Oriana
Tabopda, Turibio K.
Tabrizi , Mojgan Aghazadeh
Tachibana, Shinjiro
Tachikawa, Masanori
Tadić , Vanja
Tafi, Andrea
Taghibiglou, Changiz
Tago, Megumi
Tai, Dar-Fu
Taing, Meng-Wong
Taira, Junsei
Takada, Hideshige
Takagaki, Atsushi
Takahama, Umeo
Takahashi, Daisuke
Takahashi, Hideyo
Takahashi, Nobuyuki
Takahashi, Shigehiro
Takahashi, Wataru
Takakura, Shoji
Takamori, Kenji
Takanami, Toshikatsu
Takase-Yoden, Sayaka
Takaya, Naoki
Takayama, Fusako
Takeda, Minoru
Takyar, Seyedtaghi
Taly, Antoine
Tamariz, Joaquín
Tan, Cai-Ping
Tan, Dun-Xian
Tan, Jian-wen
Tanaka, Junichi
Tanaka, Mitsuru
Tanaka, Naonobu
Tanaka, Shigenori
Tanaka, Takuji
Tanase, Stefania
Tang, Dingqin
Tang, Jing
Tang, Jun
Tang, Qizhu
Tang, Wei
Tang, Yu
Tang, Yu-Ping
Taniguchi, Nobukazu
Tanimori, Shinji
Tanimoto, Hiroki
Tanner, John J.
Tanokura, Masaru
Tantillo, Dean
Tapscott, Stephen J.
Tarantino, Giovanni
Targett-Adams, Paul
Tashima, Kimihito
Tashima, Toshihiko
Tateno, Hiroaki
Tato, José Vázquez
Tattini, Massimiliano
Taurin, Sebastien
Tavakol, Elahe
Tayebati, Seyed Khosrow
Taylor, Alan G.
Taylor, Christopher D.
Taylor, Dennis
Teesdale-Spittle, Paul
Teixeira, Ana
Temml, Veronika
Temussi, Piero A.
Tenbrock, Klaus
Teng, Lesheng
Teng, Yuan Wen
Teodoro, Jose G.
Terrier, Nancy
Tesmer, John J.G.
Tester, Richard
Tharakaraman, Kannan
Thiel, Kristina
Thiéry, Valérie
Thomas III, Samuel W.
Thomas, Jim
Thomas, Olivier
Thomas, Olivier P.
Thomas, Wayne
Thomma, Bart
Thomson, Leonor
Thoo-Lin, Paul Kong
Tian, Lining
Tian, Shi-kai
Tian, Yupeng
Tietjen, Ian
Tilden, Andrea R.
Tischler, Dirk
Tite, Teddy
Titz, Alexander
Tiwari, Amit
Tiwari, Vaibhav
To, Ngai
Todeschini, Roberto
Tofani, Daniela
Tohno, Masanori
Tokars, Valerie L.
Tokitoh, Norihiro
Tokumura, Masahiro
Tokuyama, Shogo
Tolmachev, Vladimir
Tomaiuolo, Giovanna
Tomalia, Donald
Tomasino, Elisabeth
Tomatsu, Shunji
Tomi, Félix
Tominaga, Masahide
Tomofuji, Takaaki
Tonelli, Alan E.
Tong, Xinli
Top, Siden
Topczewski, Joseph J.
Tori, Motoo
Török, Marianna
Torras, J.
TORRENS, Francisco
Tortosa, Mariola
Tournier, Nicolas
Tousch, Didier
Toydemir, Gamze
Toyota, Shinji
Tranchida, Peter
Trapalis, Christos C.
Trent, John O.
Trifunović, Srećko R.
Trindade, Alexandre
Trindade, Reginaldo Almeida da
Tripodi, Marco
Tron, Gian Cesare
Trouw, Leendert A.
Trujillo, Cristina
Truong-Bolduc, Que Chi
Tsai, Din pin
Tsai, Tsung-Yu
Tsai, Ying Chieh
Tsakalof, A
Tsang, Hector W.H.
Tsang, Siu Wai
Tsantarliotou, Maria P.
Tsantili-Kakoulidou, Anna
Tschierske, Carsten
Tsenkova, Roumiana
Tsigenopoulos, Constantinos
Tsipis, Athanassios C.
Tsopmo, Apollinaire
Tsotsis, Theodore T.
Tsubaki, Shuntaro
Tsukamoto, Takashi
Tsurkan, Mikhail V.
Tsuruoka, Takaaki
Tsuruya, Kazuhiko
Tsutsui, Shigeyuki
Tsuyoshi, Taniguchi
Tubaro, Cristina
Tuccinardi, Tiziano
Tufarelli, Vincenzo
Tuffery, Pierre
Tung, Kwong-Chung
Turks, Māris
Turnbull, W. Bruce
Turner, Helen
Tysoe, Eddy
Tzang, Bor-Show
Ueno, Yoshihito
Ulatowski, Lynn
Ullah, Aman
Umemura, Myco
Umeyama, Akemi
Unkefer, Clifford J.
Ünsal-Tan, Oya
Upadhyay, Atul
Urayama, Paul K.
Urban, Milan
Urbieta, María Tabernero
Urbina, Julio
Urpi-Sarda, Mireia
Urszula, Kasprzykowska
Usami, Yoshihide
Usui, Kazuteru
Vaca, Inmaculada
Valappil, Sabeel P.
Vale, Nuno
Valencia, Gregorio
Valero, Manuel
Valiante, Vito
Valkunas, Leonas
Vallarino, Jose G.
Valles, Soraya L.
Vallini, Giovanni
Valterova, Irena
Van Aerschot, Arthur
Van Berkel, Willem
Van Breda, Simona
Van de Voorde, Marcel H.
Van Der Auweraer, M.
Van Der Hooft, Justin
Van der Vaart, Arjan
Van Der Veken, Pieter
Van Griensven, Leo J.L.D
Van Horn, Wade
Van Lanen, Steven
Van Opstaele, Filip
Van Otterlo, Willem A.L.
Van Sint Annaland, Martin
Vandamme, Dries
Vanderlei de Souza, Maria de Fátima
Vanderleyden, Jos
VandeVondele, Joost
Vanelle, Patrice
Vankayalapati, Hariprasad
Varela-López, Alfonso
Varela-Ramirez, Armando
Vasdev, Neil
Vasil, Michael L.
Vašková, Janka
Vásquez, Claudio C.
Vaz, Deisi Altmajer
Vázquez, Julio
Vázquez-Flota, Felipe
Vecsernyes, Miklos
Vedernikov, Andrei N.
Velasco, Pablo
Velazquez, Carlos
Velázquez, Sonsoles
Velez, Zélia
Velu, Sadanandan E
Venditti, Alessandro
Venkatachalam, T.K.
Venukadasula, Phanindra
Vepsäläinen, Jouko
Vercaigne-Marko, Dominique
Verde, Ignacio
Verma, Sanny
Vernekar, Sanjeev Kumar
Versari, Andrea
Vetvicka, Vaclav
Viappiani, Cristiano
Vicario, Jose Luis
Vicente, Ana
Vidossich, Pietro
Vieira, Margarida C.
Vieira, Mónica
Viejo-Borbolla, Abel
Vigier, Karine
Vihinen, Mauno
Viladomat, Francesc
Vilanova, Mar
Vilar, Vítor J.P.
Vilhelmsen, Mie H.
Villa, Federica
Villaño, Débora
Villoutreix, Bruno
Viñas, Miguel
Vincenzi, Simone
Vinogradov, Evgeny
Viollet, Benoit
Vioux, André
Viruel, Maria Angeles
Visioli, Francesco
Vistoli, Giulio
Viswanathan Bhagavathy, Ganga
Vitale, Alessandra
Vitorino, Rui
Viuda-Martos, Manuel
Vlase, Laurian
Voinovich, Dario
Volpi, Nicola
Von Schwarzenberg, Karin
von Zezschwitz, Paultheo
Vorsa, Nicholi
Voutquenne-Nazabadioko, Laurence
Vuong, Quan V.
Wagenknecht, Hans-Achim
Wagner, Gerd K.
Waksmundzka-Hajnos, Monika
Wallinder, Charlotta
Wallis, Russell
Walls, Zac
Walsby, Charles J.
Walter, Michael H.
Walther, Dirk
Wan, Jian-bo
Wang, Baomin
Wang, Bo-Cheng
Wang, Ce
Wang, Ching-Chiung
Wang, Chin-Kun
Wang, Chunjiang
Wang, Chunming
Wang, Chunru
Wang, Cunde
Wang, De-Yi
Wang, Erkang
Wang, Fang
Wang, Fengshan
Wang, Hao-yang
Wang, Huadong
Wang, Jeh-jeng
Wang, Junjian
Wang, Lan-ying
Wang, Liming
Wang, Liya
Wang, Peng
Wang, Qun
Wang, Robert Y.L.
Wang, Runling
Wang, S.-W.
Wang, Shaoyun
Wang, Sheng-Yang
Wang, Steven S.-S.
Wang, Tsung-Shing
Wang, Wentian
Wang, Xiang Simon
Wang, Xiangke
Wang, Xiaoping
Wang, Xiaowei
Wang, Xiaowu
Wang, Xin
Wang, Xin-Luan
Wang, Xu
Wang, Ying
Wang, Yong
Wang, Yonghua
Wang, Yongsheng
Wang, Youping
Wang, Youwei
Wang, Yue-Hu
Wang, Yun
Wang, Zhenxin
Wang, Zhezhi
Wang, Zuankai
Ward, Dale E.
Wardell, James Lewis
Watanabe, Eiki
Watanabe, Kazuhito
Watanabe, Ryuichi
Waterman, Carrie
Watson, Aaron M.
Watson, David G.
Watson, William J.W.
Webb, Andrew J.
Weber, Christine
Weber, Fabian
Weber, G.
Weekes, Ashley Ann
Węgrzyn, Grzegorz
Wei, Gang
Wei, Guor-Jien
Wei, Zhiyong
Weinhold, Frank
Weiss, Julia
Weller, Michael G.
Welters, Hannah J.
Wendell, Stacy Gelhaus
Weng, Ching-Feng
Weng, Jing-Ru
Wenjun, Wu
Wesolowski, Marek
West, James D.
West, Thomas P.
Westcott, Stephen A.
Westman, Gunnar
Westwell, Andy
Wettig, Shawn D.
Wheeler, Kraig A.
White, Mark G.
Whitehead, Saffron
Whittaker, Michael R.
Whittaker, Steven
Wiczkowski, Wieslaw
Wierzejewska, M.
Wiese, Stefan
Wiesner, Melanie
Wietrzyk, Joanna
Wijkander, Jonny
Wilde, Annegret
Wilde, Pete
Wilden, Jonathan
Willett, Peter
Williams, David
Williams, Jeffrey
Williams, Noelle S.
Williams, Thomas L.
Williams, Vance
Wills, Martin
Wilson, Lee
Wilson, W. David
Wink, Michael
Winkler, Jeffrey D.
Winnicka, Katarzyna
Wirth, Thomas
Wittstein, Kathrin
Wittung-Stafshede, Pernilla
Wlaz, Piotr
Włodarczyk, Maciej
Wójciak, Karolina M.
Wojciechowska, Agnieszka
Wojciechowski, A.
Wojdyło, Aneta
Wolińska, Ewa
Wolska, Krystyna I.
Wong, Edgar H. H.
Wong, Ka-Leung
Wong, Kwok-Yin
Woods, Robert
Woollins, Derek
Worley, S.D.
Wormley, Jr., Floyd L.
Wörner, Martin
Woźniakowski, Grzegorz
Wright, Clyde J.
Wu, Chi-Rei
Wu, Christine D.
Wu, Hao
Wu, Hsyueh-liang
Wu, Huaizhu
Wu, Jang-Yen
Wu, Jianyong
Wu, Jin-Yi
Wu, Li-Chen
Wu, Mianbin
Wu, Nianqiang
Wu, Quan-Xiang
Wu, Shih-Hsiung
Wu, Tao
Wu, Tung-Kung
Wu, Tzong-Yuan
Wu, Wei
Wu, Xiaofeng
Wu, Yang-Chang
Wu, yiqun
Wu, Yu-Jen
Wu, yu-shan
Wu, Yu-Tse
Wu, Zhanhong
Wu, Zhenghong
Wuertz, Karin
Wujcik, Evan
Würthwein, Ernst-Ulrich
Wylie, R. Stephen
Wynant, Niels
Xavier, Ana M.R.B.
Xavier, Nuno Manuel
Xia, Andong
Xia, Dingguo
Xia, Fan
Xia, Wenshui
Xia, Yuanzhi
Xiang, Guoqiang
Xiang, Lan
Xiang, Lianbin
Xiao, Jun-Xia
Xie, Jinghang
Xie, Peng
Xie, Qian
Xie, Shulian
Xie, Xiaoguang
Xie, Zhigang
Xin-Ru, Jia
Xiongo, Yan
Xu, Feng
Xu, Hui
Xu, Jingkun
Xu, Jun
Xu, Kui
Xu, Lin
Xu, Qian
Xu, Qiong-ming
Xu, Xianxiu
Xu, Xudong
Xu, Yan
Xuan, Li-jiang
Xuan, Zhu
Xue, Fengtian
Yaegaki, Ken
Yaghmur, Anan
Yajima, Shunsuke
Yamada, Kenichi
Yamaguchi, Yasuhiro
Yamaguchi, Yoshiki
Yamaki, Koji
Yamamoto, Hisashi
Yamamoto, Kazuo
Yamamoto, Megumi
Yamamoto, Yasunori
Yamani, Naouale El
Yamashita, Atsushi
Yamauchi, Satoshi
Yamazaki, Hiroyuki
Yamoto, Takehiko
Yan, Hong
Yan, Kai
Yan, Rulong
Yan, Xiufeng
Yan, Yijing
Yanada, Reiko
Yanase, Emiko
Yanez, Manuel
Yang, Bin
Yang, Chao-Hsun
Yang, Chi-chiang
Yang, Ding-I
Yang, Ding-Yah
Yang, Dong
Yang, Fafu
Yang, Hongshun
Yang, Jen-Chang
Yang, Li
Yang, Liang
Yang, Liangbao
Yang, Lili
Yang, Liqun
Yang, Mihi
Yang, Nan-Loh
Yang, Nianhong
Yang, Pei-Ming
Yang, Rong Sen
Yang, S.C.
Yang, Seung O.K.
Yang, Shengyong
Yang, Tae-Jin
Yang, Xiuwei
Yang, Yang
Yang, Yong-Hua
Yang, Yu-Chiao
Yang, Yu-liang
Yang, Yushe
Yang, Zhonghan
Yantasee, Wassana
Yao, Ching-Fa
Yao, Mei-Cun
Yao, Yanhua
Yao, Zhili
Yaoita, Yasunori
Yardin, Catherine
Yasukawa, Kiyoshi
Ye, Deyong
Ye, Jinxing
Ye, Sang-kyu
Yeh, Jan-ying
Yeh, Jui-Ming
Yeh, Shu-Lan
Yehuda, Shlomo
Yen, Feng-Lin
Yennamalli, Ragothaman
Yerabolu, Jayasudhan Reddy
Yeung, Ying-yeung
Yew, Wen Shan
Yi, Tao
Yiannakopoulou, Eugenia
Yin, Guowei
Yin, Hao
Yin, S.W.
Yin, Zhiwei
Yokomatsu, Tsutomu
Yokosuka, Akihito
Yokoyama, Kenichi
Yonekura, Shinichi
Yonezawa, Naoto
Yoo, Sung Jong
Yoo, Yeong-Min
Yoon, Do-Young
Yoon, Kee Dong
Yoon, Sang Sun
Yordanov, Yordan
Yoshida, Masaki
Yoshida, Suguru
Yoshikawa, Masaaki
Yoshimura, Yuichi
You, Liang
Young, Howard S.
Yu, Cheng-Chia
Yu, Deng-Guang
Yu, Guipeng
Yu, Hongmei
Yu, Hui
Yu, Shi-shan
Yu, Ting
Yu, Wenquan
Yu, Wing-Yiu
Yu, Xiaoqi
Yu, Yinghua
Yu, Yongxin Jeremy
Yu, Yuan-Hsiang
Yu, ZhiLing
Yu, Zhi-xiang
Yuan, C.S.
Yuan, Wei
Yuan, Yaxia
Yuasa, Hideya
Yuba, Eiji
Yue, PatrickYingKit
Yui, Tatsuto
Yum, J.H.
Yun, Sang-Seon
Zabaras, Dimitrios
Zabetakis, Ioannis
Zablackis, Earl
Zacharias, Martin
Zago, Miriam
Zähringer, Ulrich
Zajac, Adrian
Zakarian, Armen
Zakrzewicz, Dariusz
Zalacain, Amaya
Zamora, Fernando
Zanotti, Gloria
Zarrelli, Armando
Zawilska, Jolanta B.
Zehl, Martin
Zeisel, Mirjam B.
Zelkó, Romána
Zeng, Huaqiang
Zeng, Jie
Zeng, Minghua
Zeng, Xiaoming
Zeng, Xin-An
Zeni, Gilson
Zervoudakis, George
Zhang, Dayi
ZHANG, Deqing
Zhang, Dong-ming
Zhang, Ge
Zhang, Guolin
Zhang, Hongyu
Zhang, Hu
Zhang, Huiming
Zhang, Jian
Zhang, Jianzhong
Zhang, Jingyan
Zhang, Jinsong
Zhang, Ji-Wen
Zhang, Junbo
Zhang, Junzeng
Zhang, Lin
Zhang, Liping
Zhang, Longhe
Zhang, Mengliang
Zhang, Mingwei
Zhang, Qing
Zhang, Qingwen
Zhang, Shaozhong
Zhang, Shu Fen
Zhang, Suoqin
Zhang, Tong-lai
Zhang, Wei
Zhang, Wenheng
Zhang, Xian-fu
Zhang, Xiang
Zhang, Xiao-Bing
Zhang, Xiaohong
Zhang, Xiaoyun
Zhang, Xinyu
Zhang, Xiurong
Zhang, Xue-Jun
Zhang, Yunwu
Zhang, Zhenfeng
Zhao, Gui-sen
Zhao, Guohua
Zhao, Han
Zhao, Hongtao
Zhao, Hong-Wu
Zhao, Hua
Zhao, Jian-She
Zhao, Jianzhang
Zhao, Jin
Zhao, Jin-Hao
Zhao, Liang
Zhao, Ming
Zhao, Qinshi
Zhao, Xin-Qing
Zhao, Yanchuan
zhao, yilei
Zhao, Ying-Yong
Zhao, Yuxia
Zhen, Xuechu
Zheng, Gao-Wei
Zheng, Qi-Huang
Zheng, Shilong
Zheng, TianLing
Zheng, Yanjie
Zhong, Chen
Zhong, Haizhen A.
Zhou, Aimin
Zhou, C.R.
Zhou, Guangyuan
Zhou, Guohua
Zhou, Jian
zhou, Jie
Zhou, Ligang
Zhou, Yifa
Zhou, Zhong-mei
Zhu, Guonian
Zhu, Xiaofeng
Zhu, Yinghuai
Zhuravlev, Fedor
Zielinska, Danuta
Zielińska, Sylwia
Zielińska-Pisklak, Monika A.
Zigon, D.
Ziv, Ilan
Złotek, Urszula
Zoidis, Evangelos
Żołnowska, Beata
Zong, Xuxiao
Zorc, Branka
Zou, Xiaobo
Zoumpoulakis, Panagiotis
Zucca, Antonio
Zuhorn, Inge S.
Zuñiga, Gustavo
Zuo, Yuegang
Zuo, Hua
Zuykov, Michael
’t Hoen, Peter

